# Tuning the Surface Properties of Poly(Allylamine Hydrochloride)-Based Multilayer Films

**DOI:** 10.3390/ma14092361

**Published:** 2021-05-01

**Authors:** Justyna Ciejka, Michal Grzybala, Arkadiusz Gut, Michal Szuwarzynski, Krzysztof Pyrc, Maria Nowakowska, Krzysztof Szczubiałka

**Affiliations:** 1 Department of Engineering and Technology of Chemical Processes, Faculty of Chemistry, Wroclaw University of Science and Technology, Wybrzeze Wyspianskiego 27, 50-370 Wroclaw, Poland; 2 Department of Physical Chemistry and Electrochemistry, Faculty of Chemistry, Jagiellonian University, Gronostajowa 2, 30-387 Krakow, Poland; arkadiusz.gut@uj.edu.pl (A.G.); nowakows@chemia.uj.edu.pl (M.N.); 3 Virogenetics Laboratory of Virology, Malopolska Centre of Biotechnology, Jagiellonian University, Gronostajowa 7A, 30-387 Krakow, Poland; k.a.pyrc@uj.edu.pl; 4 Department of Microbiology, Faculty of Biochemistry, Biophysics and Biotechnology, Jagiellonian University, Gronostajowa 7, 30-387 Krakow, Poland; michal.grzybala@alumni.uj.edu.pl; 5 Academic Centre for Materials and Nanotechnology, AGH University of Science and Technology, Mickiewicza 30, 30-059 Krakow, Poland; szuwarzy@agh.edu.pl

**Keywords:** poly(allylamine hydrochloride), layer-by-layer, polyelectrolytes, hydrophilic, amphiphilic, polyelectrolyte multilayer

## Abstract

The layer-by-layer (LbL) method of polyelectrolyte multilayer (PEM) fabrication is extremely versatile. It allows using a pair of any oppositely charged polyelectrolytes. Nevertheless, it may be difficult to ascribe a particular physicochemical property of the resulting PEM to a structural or chemical feature of a single component. A solution to this problem is based on the application of a polycation and a polyanion obtained by proper modification of the same parent polymer. Polyelectrolyte multilayers (PEMs) were prepared using the LbL technique from hydrophilic and amphiphilic derivatives of poly(allylamine hydrochloride) (PAH). PAH derivatives were obtained by the substitution of amine groups in PAH with sulfonate, ammonium, and hydrophobic groups. The PEMs were stable in 1 M NaCl and showed three different modes of thickness growth: exponential, mixed exponential-linear, and linear. Their surfaces ranged from very hydrophilic to hydrophobic. Root mean square (RMS) roughness was very variable and depended on the PEM composition, sample environment (dry, wet), and the polymer constituting the topmost layer. Atomic force microscopy (AFM) imaging of the surfaces showed very different morphologies of PEMs, including very smooth, porous, and structured PEMs with micellar aggregates. Thus, by proper choice of PAH derivatives, surfaces with different physicochemical features (growth type, thickness, charge, wettability, roughness, surface morphology) were obtained.

## 1. Introduction

Surface modification is one of the strategies used to tailor the properties of objects, with sizes spanning several orders of magnitude, to make them suitable for practical applications. Such modification may change the chemical, biological, physical properties of the materials, allowing their use, e.g., as drug-delivery systems, cosmetics, textiles, adsorbents, membranes, self-healing and anticorrosive materials, to mention just a few examples [1,2,3].

The layer-by-layer (LbL) technique is a well-established method of surface modification in the nanoscale, introduced already in the 1960s by Iler [4] and later developed by Decher [5]. Pairs of oppositely charged polymers (polyelectrolytes) are particularly suitable for this method because of their ability to form polyelectrolyte multilayers (PEMs) on any charged surface due to the strong electrostatic attraction. Polymers used for PEM formation may be both synthetic and (modified) natural polymers. The most often studied synthetic polyanions include poly(acrylic acid) (PAA) [6,7,8], poly(methacrylic acid) (PMA) [9,10], and poly(sodium 4-styrenesulfonate) (PSSS) [11,12,13,14,15,16,17,18,19,20], while the typical synthetic polycations are poly(allylamine hydrochloride) (PAH) [6,8,17,19,21,22], polyethyleneimine (PEI) [7], poly-l-lysine (PLL) [18,23,24,25,26], and poly(diallyldimethylammonium chloride) (PDADMAC) [11,12,14,15,17]. On the other hand, (modified) natural polymers include chitosan [10,27,28,29,30], dextran derivatives [31], hyaluronic acid [18,23,24,26,27,28], and alginate [10,27].

A great advantage of the LbL method of PEM fabrication is that practically any pair of oppositely charged polyelectrolytes can be used. Therefore, in a vast majority of PEMs studied so far, both polymers are of a different chemical nature, including various molecular weights, dispersity indices, backbone architecture, chain flexibility, etc. Consequently, it may be difficult to ascribe a particular physicochemical property of the resulting PEM (e.g., exponential vs. linear growth, topology of the surface, wettability, etc.) to a single structural or chemical feature of the component polymers. A solution to this problem may be the application of a polycation and a polyanion obtained by proper modification of the same parent polymer. By using the derivatives of the same polymer as polycations and polyanions for the PEM construction it may be possible to ascribe the differences in their properties solely to the differences in substituting groups and to exclude the role of the differences in the properties of the polymeric backbones, e.g., structural stiffness.

PEMs obtained from two oppositely-charged derivatives of the same polymer have already been reported, both for synthetic polymers, e.g., silicones [32] or PAH [33,34] and a natural polymer, i.e., chitosan [29,35]. However, these studies included PEMs obtained using just one pair (two in one case [33]), so they did not allow drawing conclusions on the influence of polymer substitution on the PEM behavior.

This paper is, to our best knowledge, the first systematic study on novel PEMs formed by several strong polyelectrolytes obtained by anionic and cationic modification of the same polymeric chain. As the parent polymer we chose PAH, a commercially available, inexpensive polymer with simple and flexible hydrocarbon backbone. The reactive amine groups of PAH could be easily substituted with hydrophobic hexyl groups, charged (sulfonic or trimethylammonium groups of glycidyltrimethylammonium chloride (GTMAC)), or quaternized into trimethylammonium cations, so PAH could be transformed from a weak polyelectrolyte into several strong (amphiphilic) polyelectrolytes forming six different PEMs.

## 2. Materials and Methods

### 2.1. Materials

Poly(allylamine hydrochloride) (PAH, Mw ~17.5 kDa), N-methyl-2-pyrrolidone (NMP, ≥99%), iodomethane (99%), potassium iodide (≥99%), sodium chloride (≥99.5%), sodium hydroxide (≥98%), glycidyltrimethylammonium chloride (GTMAC, ≥90%), sodium borohydride (NaBH4), hexanal (98%), sulfur trioxide-trimethylamine complex (TMAS), dimethyl sulfoxide-d6 (99.9% D), deuterium oxide (99.9% D), and tetraethyl orthosilicate (TEOS, 98%) were obtained from Sigma-Aldrich (MERCK, Poznań, Poland). Glacial acetic acid (≥99.85%), hydrochloric acid (36.5–38%), methanol, ethanol, potassium carbonate, and sodium carbonate were obtained from Polish Chemical Reagents (POCh, Gliwice, Poland). Deionized water was used in all experiments. Dialyses were conducted using dialysis tube (T1, MWCO 3.5 kDa) obtained from ZelluTrans/Roth T1 (Linegal Chemicals, Warszawa, Poland).

### 2.2. Synthesis and Characterization of Polymers

#### 2.2.1. N-Methylated Poly(Allylamine) (C1H)

**Scheme 1:**
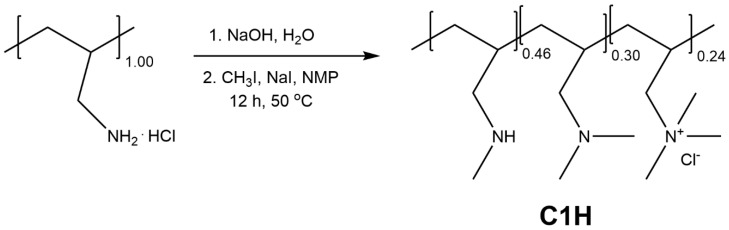
Synthesis of C1H.

The synthesis of poly(allylamine hydrochloride) (PAH) substituted with methyl groups, i.e., N-methylated poly(allylamine) (C1H) was conducted according to a modified protocol reported by Wytrwal et al. [34] (Scheme 1).

Briefly, PAH (1.5 g, Mw 17.5 kDa, 16.03 mmol of amine groups) was dissolved in 20 mL of water and then treated with sodium hydroxide (0.86 g, 21.5 mmol). The solution was suspended in 80 mL of N-metyl-2-pyrrolidone (NMP) and stirred for 30 min at room temperature. Subsequently, potassium iodide (2.8 g, 16.9 mmol) and iodomethane (6 mL, 128.5 mmol, 8-fold excess vs. PAH amine groups) were added. The reaction mixture was stirred for 12 h at 50 °C. Then, the solution was cooled down and transferred to the dialysis tube. The dialysis was carried out first against water (5 days) then against 0.1 M aqueous potassium chloride (3 days, in order to exchange iodide anions into chloride anions), and again against water (4 days). The excess of solvent was removed under reduced pressure and then the product C1H was isolated from the solution by freeze-drying technique to obtain a pale yellow crystalline product (1.17 g, 62%). The methylation degree of amine groups was estimated by means of nuclear magnetic resonance (^1^H NMR, Figure S2 in Supplementary Material). Deconvolution of the overlapping signals of the methyl groups enabled calculation of the degree of substitution DS values which were found to be 46, 30, and 24% for mono-, di-, and trimethylated amine groups, respectively.

#### 2.2.2. Poly[(3-Allylamino-2-Hydroxypropyl)Trimethylammonium Chloride] (C2H)

**Scheme 2:**
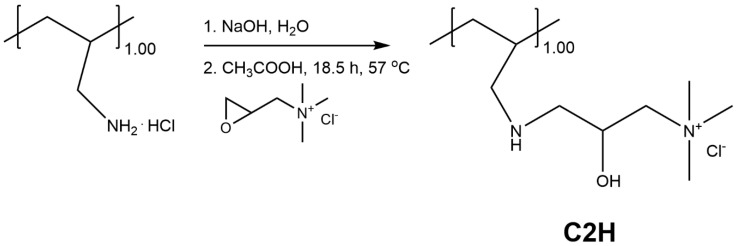
Synthesis of C2H.

PAH substituted with GTMAC, i.e., poly[(3-allylamino-2-hydroxypropyl)-trimethylammonium chloride] (C2H) was synthesized using a modified procedure reported elsewhere [34] (Scheme 2).

PAH (1.5 g, 17.5 kDa, 16.03 mmol of amine groups) was dissolved in 55 mL of water. Subsequently, 0.84 g of sodium hydroxide (21.0 mmol) was added to the PAH solution and stirred for 30 min. After this time, the solution was neutralized by dropwise addition of 10% HCl until pH = 7 was reached. Then, a catalytic amount of acetic acid (0.38 mL of 0.5% *v/v* CH_3_COOH) was added. The mixture was stirred for another 30 min at room temperature, then GTMAC (≥90%, 20 mL, 134–149 mmol) was added dropwise while stirring. The reaction was continued for 18.5 h at 57 °C. Upon cooling down, the reaction mixture was transferred to the dialysis tube and dialyzed against distilled water for 12 days. The solution was concentrated under reduced pressure and then freeze-dried to obtain colorless solid (2.63 g, 78%). DS was 100% as found from ^1^H NMR spectrum (Figure S3 in Supplementary Material).

#### 2.2.3. Poly{[(Allyltrimethylammonium Chloride)*-co-*(3-Allylamino-2-Hydroxy-Propyl)Trimethylammonium Chloride]*-co-*[N-Allyl-N-Hexylamine]} (CAm)

**Scheme 3:**
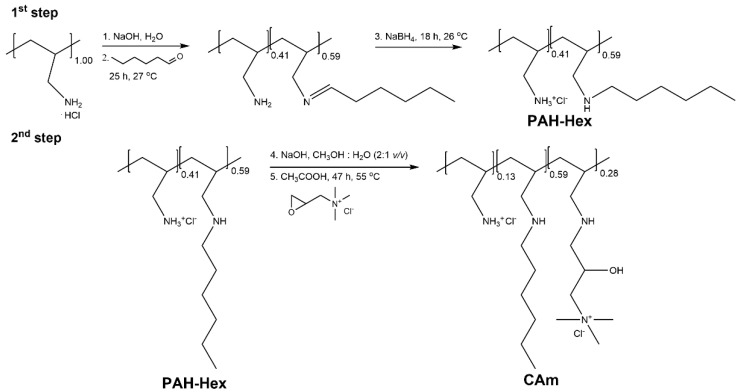
Synthesis of amphiphilic polycation (CAm).

A novel positively charged amphiphilic derivative of PAH, i.e., poly{[(allyltrimethylammonium chloride)*-co-*(3-allylamino-2-hydroxypropyl)trimethyl-ammonium chloride]*-co-*[N-allyl-N-hexylamine]} (CAm) was synthesized via a two-step route (Scheme 3).

In the first step the amine groups of PAH were substituted with n-hexyl chains by imine formation followed by the reduction with sodium borohydride. Then, non-substituted amine groups were reacted with GTMAC to obtain a water-soluble amphiphilic polycation (CAm).

The first step of the synthesis was carried out according to the modified method previously reported for chitosan by Jia et al. [36] PAH (1.0 g, 17,5 kDa, 10.69 mmol of amine groups) was dissolved in 8 mL of water. Subsequently, 2 mL of 2.5 M NaOH (5 mmol) was carefully added. Then, the mixture was acidified using 2% HCl until pH reached 3.6. 3.28 mL of hexanal (26.69 mmol, 2.5-fold excess vs. PAH amine groups) was added dropwise to the reaction mixture and stirred in a closed vessel for 25 h at 27 °C. Then, 2.54 g of NaBH_4_ (67.14 mmol) dissolved in 3 mL of water was carefully added. Caution is required: a solution of NaBH_4_ has to be slowly dropped into the reaction mixture to avoid excessive foaming. The reduction reaction was carried out for 18 h at 26 °C. After this time, the suspension formed was transferred to the dialysis tube and dialyzed against water for 12 days. Then, the dialysate was exchanged for 0.1 M HCl and the process was continued for 1 h, with the subsequent dialysis against water for 5 days. The product (PAH–Hex) was isolated by lyophilization to obtain 1.31 g of white solid. The degree of substitution with n-hexyl groups in PAH–Hex was estimated at 59% based on elemental analysis (see Section 2.4.1).

Subsequently, 330 mg of PAH–Hex (0.006 mmol of free amine groups) was dissolved in 3 mL of methanol and diluted with distilled water (2:1, *v/v*). The opalescent solution was neutralized using NaOH. Then, 0.6 mL of 0.5% acetic acid was added and stirred for 15 min before addition of 1.5 mL of GTMAC (≥90%, 1.5 mL, 10.06–11.18 mmol). The reaction was carried out for 47 h at 55 °C. After this time, the opalescent mixture was transferred to dialysis tube and dialyzed against water for 6 days. The excess of the solvent was removed under reduced pressure and the product was freeze-dried to obtain 275 mg of CAm as a pale yellow solid. The product was highly hygroscopic and was stored in a desiccator. The content of unsubstituted, n-hexyl substituted, and GTMAC-modified amine groups was calculated from elemental analysis (see Section 2.4.1) and deconvolved ^1^H NMR spectrum (see Figure S4 in Supplementary Material) to be 13, 59, and 28%, respectively.

#### 2.2.4. N-Sulfonated Poly(Allylamine Hydrochloride) (AH)

**Scheme 4:**
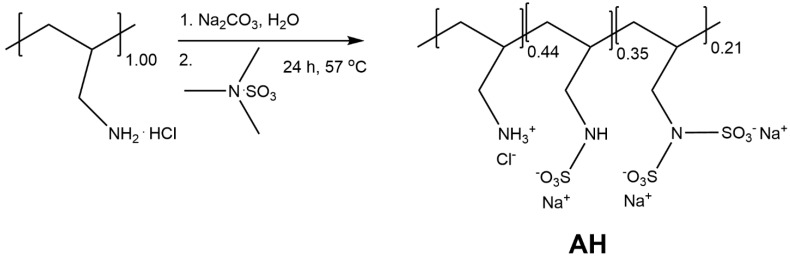
Synthesis of polyallylamine (AH).

Sulfonation of PAH to obtain N-sulfonated polyallylamine (AH) was carried out according to the modified method of chitosan sulfonation reported by Holme et al. [37] (Scheme 4).

Briefly, PAH (1.5 g, 17.5 kDa, 16.03 mmol of amine groups) was dissolved in 75 mL of water and then treated with sodium carbonate (3.5 g, 33.02 mmol, 2-fold excess vs. PAH amine groups) and mixed for 30 min until complete dissolution of the salt. After this time, sulfur trioxide–trimethylamine complex (TMAS, 6.7 g, 48.14 mmol, 3-fold excess vs. PAH amine groups) was added and the reaction was allowed to stir for 24 h at 57 °C with constant purging with nitrogen. The reaction mixture was cooled down and transferred to the dialysis tube and dialyzed against water for 4 days. The solution was concentrated under reduced pressure and freeze-dried to form a light yellow product (2.01 g, 83%). The sulfonation degree was estimated by means of ^1^H NMR. The content of unsubstituted, mono- and disubstituted amine groups was found to be 44%, 35%, and 21%, respectively, based on deconvoluted ^1^H NMR spectrum (Figure S5 in Supplementary Material).

#### 2.2.5. Poly{[(N-Sulfonyl)Allylamine]*-co-*[(N-Allyl-N-Hexylamine]*-co-*[(N-Allyl-N-Hexyl-N-Sulfonylamine]} Hydrochloride (AAm)

Novel negatively charged amphiphilic derivative of PAH was synthesized via a two-step route. The first step of the synthesis was the same as in the synthesis of CAm described above. In the second step, 325.75 mg of PAH-Hex (0.006 mmol of free amine groups) was dissolved in methanol and diluted with distilled water (*v/v* 1:6). Then, 498.94 mg of potassium carbonate (3.6 mmol) was added, and an opalescent solution was formed. The mixture was heated at 60 °C and 982.89 mg of sulfur trioxide - trimethylamine complex (TMAS, 7.06 mmol) was added. The reaction was carried out for 24 h with constant stirring and purging with nitrogen. The mixture was cooled down and transferred to a dialysis tube. The crude product was dialyzed against water for 9 days. Dialyzed solution was then concentrated under reduced pressure and freeze-dried to obtain 380.93 mg of colorless product AAm (poly{[(N-sulfonyl)allylamine]*-co-*[(N-allyl-N-hexylamine]-*co*-[(N-allyl-N-hexyl-N-sulfonylamine]} hydrochloride). The content of (N-sulfonyl)allylamine, N-allyl-N-hexylamine, and N-allyl-N-hexyl-N-sulfonylamine units was calculated using elemental analysis (see Section 2.4.1) and deconvolved ^1^H NMR spectrum (Figure S6 in Supplementary Material) to be 41%, 25%, and 34%, respectively.

### 2.3. Preparation of Polyelectrolyte Multilayers (PEMs)

PEMs composed of the synthesized PAH-based polymers were prepared on silicon wafers (1 × 1 cm). Prior to PEM assembly, the silicon wafers were treated with “piranha” solution (mixture of 36.5% sulfuric acid and 30% hydrogen peroxide, 1:1 *v/v*) for 30 min, rinsed exhaustively with distilled water, and dried with nitrogen. PEMs were prepared via the dip coating method using 1 mg/mL polymer solutions in 0.1 M NaCl [29]. The process of polymer deposition on the substrate surface started with the deposition of a polycation on the negatively charged silicon surface. The wafers were incubated for 30 min in a given polymer solution, then rinsed thrice with distilled water and dried with nitrogen. The procedure was repeated until 15 layers were obtained.

### 2.4. Physicochemical Characterization of the Poly(Allylamine Hydrochloride) (PAH) Derivatives and Their PEMs

#### 2.4.1. Elemental Analysis and Spectroscopic Methods

The analysis of elemental composition (C, H, N, and S) of freeze-dried samples (see Supplementary Material) was performed with an EuroEA 3000 Elemental analyzer. Fourier transform attenuated total reflection infrared (FT-ATR-IR) spectra (Figure S1) of solid polymer samples were recorded with IR Nicolet IR200FT-IR spectrophotometer equipped with an ATR accessory (Thermo Scientific). ^1^H NMR spectra (Figures S2–S6) were recorded with a Bruker Avance III HD 400 MHz or Bruker Avance III 600 MHz spectrometer (both Bruker, Billerica, USA) in D_2_O or DMSO-d6. Deconvolution of spectra was performed by Gauss function fitting using MestReNova software. Function width was constrained at 0.25–5.00 Hz.

#### 2.4.2. Zeta Potential

Zeta potential measurements were performed at 22 °C using the Zetasizer Nano ZS apparatus (Malvern Instruments, Worcestershire, UK). Samples of polymers (1 mg/mL) were measured in 0.015 M NaCl by using laser Doppler velocimetry and analyzed using the software provided by the manufacturer. Reported ζ are mean values from five measurements.

#### 2.4.3. Ellipsometry

For ellipsometric measurement, PEMs were prepared according to above described procedure using 1 × 1 cm silicon wafers. After each layer deposition, thickness and optical constants of dry PEMs were measured using a variable angle spectroscopic ellipsometer M-2000U (J. A. Woollam, Lincoln, USA) equipped with the Compete EASE program for data analysis. Prior to deposition of the PEM films, the thickness of oxide layer on the silicon substrate was measured. Measurements were carried out at three angles of incidence, i.e., 60, 65, and 70°. The working region of the wavelength was set to 250–1000 nm, however, to avoid the influence of absorption in the ultraviolet region by amphiphilic polymers, the analysis was carried out in the wavelength range of 400–1000 nm. All measurements were carried out in air at room temperature. To fit ellipsometric data obtained for wet films, a three-layer model was used, in which the first two layers was silicon substrate and silicon oxide layer (resulted from incubation in “piranha” solution) and the third layer was the PEM film. The polymer layer was treated as a Cauchy material of thickness having a wavelength-dependent refractive index n(λ) = A + B/λ^2^ + C/λ^4^, where A, B and C are fitting coefficients and λ is the wavelength. The film extinction coefficient was assumed to be negligible (k = 0). The thickness, d, and the three coefficients A, B, and C were fitted simultaneously. Presented data are the means of three independent measurements with standard deviation.

#### 2.4.4. PEM Wettability

Water contact angles of PEMs were measured by the sessile drop method using Surftens Universal instrument (OEG GmbH, Frankfurt, Germany). PEMs were deposited on silicon wafers (1 × 1 cm) as described above. A water droplet was deposited on the surface of the tested PEM and the image was captured using a CCD camera. The shape of water droplet was analyzed using Surftens 3.0 software to calculate the contact angle of both sides of the droplet. The measurements were carried out at room temperature. The data were the mean values from three measurements of the droplet deposited on two independent samples.

#### 2.4.5. Atomic Force Microscopy (AFM)

The topography images of the surface of the PEMs coated on silicon wafers were obtained using a Dimension Icon atomic force microscope (AFM, Bruker, Santa Barbara, CA, USA) atomic force microscope in the PeakForce Tapping (PFT) mode with the standard silicon cantilevers of normal spring constant of 0.7 N/m and tip radius of 2 nm. All measurements were carried out after 30 min incubation in phosphate-buffered saline (PBS), 1 M sodium chloride solution or using dry samples, with low setpoint parameters to avoid damage of soft matter samples. The thickness of the PEMs was measured under the same conditions after mechanically made scratch was obtained with Teflon tweezers.

## 3. Results and Discussion

### 3.1. General Characteristics of the Polymers

**Scheme 5:**
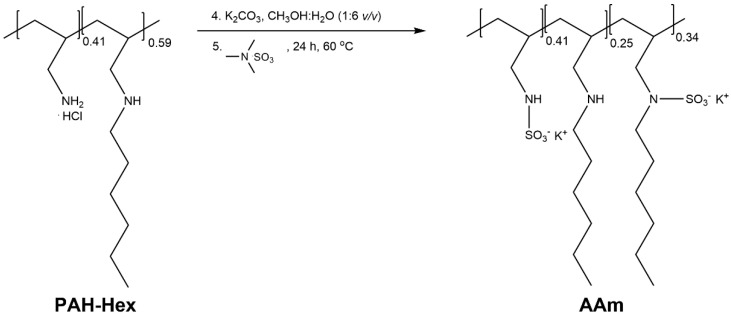
The second step of AAm (poly{[(N-sulfonyl)allylamine]*-co-*[(N-allyl-N-hexylamine]*-co-*[(N-allyl-N-hexyl-N-sulfonylamine]} hydrochloride) synthesis.

Five derivatives of PAH were obtained by substitution of its amine groups to obtain ionic (both anionic and cationic) polymers of both hydrophilic and amphiphilic character. In contrast to parent PAH, the synthesized polymers were strong polyelectrolytes since they contained either sulfonic groups (introduced by N-sulfonation of PAH with sulfur trioxide–trimethylamine complex (TMAS) [37]) or trimethylamine quaternary ammonium groups (introduced by means of quaternization of amine groups by exhaustive methylation with methyl iodide [34] or by substitution with glycidyltrimethylammonium chloride (GTMAC) [34] as confirmed by elemental analysis, spectroscopic methods (see Supplementary Material) and results obtained from surface zeta potential measurements (see below). Three of them, denoted as C1H, C2H and CAm (where C stands for “cationic”, H for “hydrophilic”, and Am for “amphiphilic”, Scheme 1, Scheme 2 and Scheme 3) were polycations, while two polymers, denoted as AH and AAm (where A stands for “anionic”, Scheme 4 and Scheme 5) were polyanions. In fact, AH was a polyzwitterion since at neutral pH it contained both anionic SO_3_^-^ and partially protonated amine groups (pKa of amine groups in PAH is 8.6 [38]); however, the content of anionic groups was much higher (77 vs. 44 mol%) giving the polymer overall negative charge. Three polymers (C1H, C2H, and AH) were highly hydrophilic, while two of them, i.e., CAm and AAm, showed amphiphilic character since they contained, on one hand, charged trimethylammonium or sulfonic groups (41 and 75 mol%, respectively) and, on the other hand, hydrophobic hexyl groups (59 mol% both, in AAm 34 mol% of the amino groups were both hexylated and sulfonated) as confirmed by elemental analysis and spectroscopic methods (see Supplementary Material). In the aqueous media their macromolecules self-assembled into pseudomicellar structures which were retained at the surface after deposition, as confirmed by AFM surface topology imaging of the PEMs (see morphology of PEMs in Section 3.2.3 and Figures S9–S10 in Supplementary Material).

The results of zeta potential measurements of the polymers are presented in Table 1. All the polymers showed high (over ±30 mV) zeta potential at neutral pH so strong electrostatic interaction between their layers in PEMs and consequently their stability could be expected.

### 3.2. Physicochemical and Morphological Characterization of PEMs

#### 3.2.1. Growth Models of PEMs

**Figure 1:**
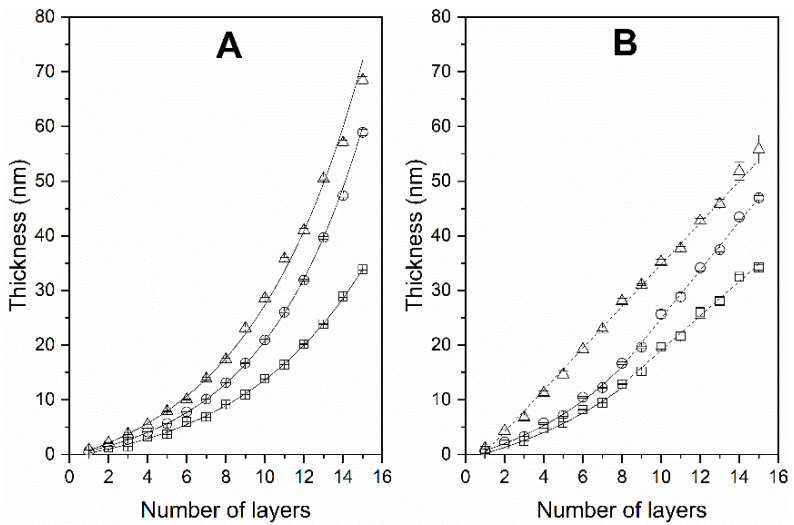
Thickness of polyelectrolyte multilayer s (PEMs) obtained using polycations: C1H (squares), C2H (circles) and CAm (triangles) alternately deposited with AH (**A**) or AAm (**B**). Solid and dashed lines indicate the regions of exponential and linear growth of film thickness, respectively. The thickness data were obtained by ellipsometric measurements after deposition of each polymer layer.

To obtain PEMs composed of up to 15 layers C1H, C2H, or CAm polycations were alternately deposited with AH or AAm polyanions so all 6 theoretically possible PEMs were obtained, i.e., C1H&AH, C1H&AAm, C2H&AH, C2H&AAm, CAm&AH, and CAm&AAm. The results obtained from spectroscopic ellipsometry measurements indicated that the thickness of PEMs differed significantly between the polymer pairs and reached 30–70 nm for 15-layer PEMs (Figure 1). Monotonous growth of film thickness suggests that dissociation of amphiphilic macromolecules from PEM surface did not occur during washing steps as it was observed for PEM coatings prepared from weak polyelectrolytes [39]. The growth of PEM was analyzed using the methodology developed earlier by C. A. Helm and co-workers [40,41].

The PEMs obtained in these studies showed three modes of thickness growth that may be attributed to the effect of different charge density of PAH derivatives and side chain hindrance during formation of PEM film and its growth.

The direct consequence of the differences in the type of growth of the layer-by-layer assemblies is the diversity in the thickness of tested coatings. Indeed, although all tested coatings were prepared in the same conditions (polymer concentration, deposition time, temperature, solvent [42]), the average thickness of 15-layer C1H&AH, C1H&AAm, C2H&AAm, CAm&AAm, C2H&AH and CAm&AH coatings, right after preparation, differed significantly and was found to be 33, 39, 50, 57, 58 and 69 nm, respectively, based on ellipsometric measurements.

It is worth mentioning here that the type of PEM growth depends mostly on the type of polyanion used as opposite layer. When a PEM contained AH as a polyanion, the exponential growth of film thickness was observed, regardless of the type of oppositely charged polymer used (Figure 1A). The exponential increase in film thickness is generally attributed to the ability of macromolecules to diffuse deep into the PEM owing to the loose film structure, in contrast to PEM films with linear growth of thickness that are compact and impermeable [43,44]. Thus, exponential growth of film thickness when hydrophilic AH polyanion is alternately deposited with tested polycations suggests that regardless of the type of oppositely charged polyelectrolyte, i.e., hydrophilic C1H and C2H or amphiphilic CAm polycations, the interlayer diffusion of polyelectrolyte chains through the PEM film is possible in the whole tested range, suggesting that the PEM structure of C1H&AH, C2H&AH and CAm&AH is rather loose. Moreover, it seems that for PEMs formed by AH the rate of thickness growth of a PEM is related to the size of the substituents in the polycation macromolecules and may be attributed to the steric hindrance that is smallest for C1H, intermediate for C2H, and largest for CAm.

Interestingly, for PEMs composed of amphiphilic AAm as a polyanion and hydrophilic C1H or C2H as polycations, i.e., C1H&AAm and C2H&AAm, the dependence of the film thickness on the layer number showed two distinct regions, with slower exponential growth up to the 8th layer and a faster linear growth from 8th up to 15th layer (Figure 1B). Such PEMs showing a change of the growth type from linear to faster exponential or vice versa were reported in literature [45]. The change from linear to exponential growth may indicate an increase in vertical mobility of the polymeric chain with increasing number of layers [43]. This may be ascribed to increased reptation of the polymeric chains through the meshes of the polymeric matrix and/or a lower number of connection points. Alternatively, based on models proposed by Hübsch et al. [19] and Salomäki et al. [46], the exponential-to-linear transition may result from film restructuring and densification which increasingly inhibits diffusion of the polyelectrolyte chains into the PEM. This forbidden area grows with the number of layers so that the outer zone of the film maintains a constant thickness. Exponential-to-linear transition was also observed for systems containing a mixture of polyelectrolytes of the same charge so that two polycations or two polyanions compete for their incorporation into the film [19]. It should be noted that the systems showing exponential to linear change of growth regime composed of one polycation and one polyanion, as in case of AAm&C1H and AAm&C2H, are quite unique. It is suggested here that short alkyl chains present in amphiphilic AAm polyanion limit macromolecular chains movement through the network of PEM coatings, and also, considering the change in kinetic growth from exponential to linear, that upper layers of PEM films become more compact and tangled, thus more durable.

Finally, the thickness growth of a PEM composed of two amphiphilic polymers, i.e., CAm&AAm, was linear almost in the whole range of layer thickness (Figure 1B triangles). This suggests that the layers of both polyelectrolytes strongly interpenetrate due to the diffusion of the polyelectrolyte chains in the direction perpendicular to the film surface, as suggested by Elbert et al. [45]. Thus, this film is the most compact and tangled among all tested films with low permeability which may be due to the micellar structure of the films [47]. This may be presumed as PEM coatings composed of at least one amphiphilic polyelectrolyte, polycation or polyanion, are rougher than the others as evidenced by higher value of C parameter in formulas of Cauchy model, and confirmed by AFM (results discussed in next sections). These results suggest that hydrophobic anchor present in both polymeric chains may inhibit their diffusion through the film not only because of higher molecular weight of amphiphilic polyelectrolytes, but also due to the formation of mixed micellar structures.

To the best of our knowledge such a variety of growth behaviors of PEMs composed of a single (PAH in our case) polymer backbone having different substituents has not been observed before. Moreover, results obtained in our studies are quite unique and can be contrasted with the Guyomard and co-authors work as they observed the exponential growth for amphiphilic carboxymethylpullulan (CMP) derivatives and linear for hydrophilic CMP [48]. The reason may be the fact that the backbone of polyanions used by them, both hydrophilic pullulan and its amphiphilic derivatives, was much stiffer than that of polyallylamine polymers, which were used in these studies.

#### 3.2.2. Wettability of the PEMs

**Figure 2:**
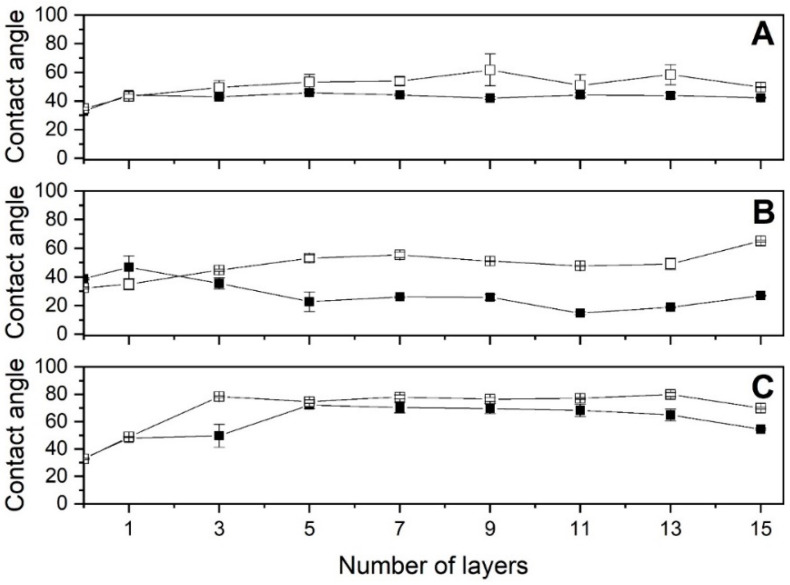
Water contact angle of PEMs with positively charged topmost layer composed of C1H (**A**), C2H (**B**) and CAm (**C**) alternately deposited with AH (black squares) or AAm (white squares).

**Figure 3:**
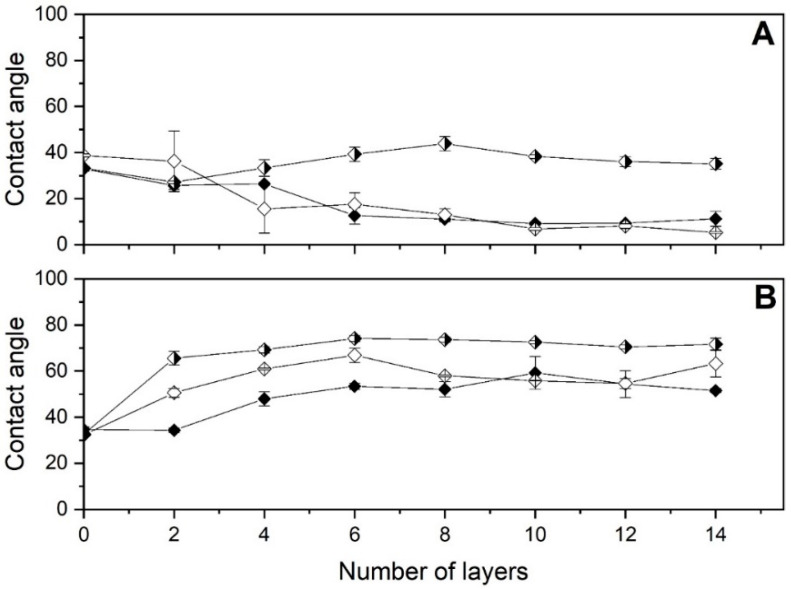
Water contact angle of PEMs with negatively charged topmost layer composed of AH (**A**) and AAm (**B**) alternately deposited with C1H (black diamonds), C2H (white diamonds), or CAm (black and white diamonds).

To study the influence of the polymer structure on the PEM wettability, the water contact angle was measured for all six PEMs obtained. The results of the measurements for PEMs with cationic (i.e., with the topmost layer composed of a polycation) and anionic (i.e., with the topmost layer composed of a polyanion) surfaces are shown in Figure 2 and Figure 3, respectively.

For the PEMs with cationic topmost layer (i.e., PEMs with odd number of layers, Figure 2) the contact angle changes after deposition of each bilayer up to 5 layers and for a higher number of layers stabilizes at an approximately constant value. For PEMs with anionic topmost layer (i.e., PEMs with even number of layers, Figure 3) contact angle stabilization is reached at the 6th layer. These changes apparently result from the substrate effect and indicate the number of layers that should be supported to obtain PEMs with well-defined wettability.

The approximate values of contact angles for PEMs composed of at least 5–6 layers are given in Table 2.

As expected, the values of contact angles for PEMs composed of polymers which are both hydrophilic, i.e., C1H&AH and C2H&AH, are lowest. Those with the topmost layer composed of AH in particular, show extremely low contact angle (about 10°) which may be ascribed to the high content of charged sulfonate groups in AH. On the other hand, greater cationic charge of C2H than that of C1H results in a lower contact angle for the respective PEMs with cationic topmost layer.

The PEMs, which are composed of one amphiphilic and one hydrophilic polymer, i.e., CAm&AH, C1H&AAm, and C2H&AAm, show intermediate hydrophilicity corresponding to contact angle values of 40–70°. Those of them containing AAm as the polyanion (i.e., C1H&AAm and C2H&AAm) show the same hydrophilicity, irrespective of the charge of the topmost layer, while for CAm&AH there is a significant difference in the contact angle (70 vs. 40°) between the PEMs with the cationic and anionic topmost layers.

Finally, the PEM composed of polymers, which are both amphiphilic, i.e., CAm&AAm, is hydrophobic, especially when cationic CAm is the topmost layer (contact angle about 80°). Furthermore, the higher roughness (see Section 3.2.3) of the AAm-alternated films in comparison to AH-alternated films surfaces may also contribute to lowering PEMs wettability because physical properties, surface energy, and surface morphology all influence water contact angle.

The contact angle values for the PEMs obtained cover the range from very hydrophilic (10°) to quite hydrophobic (80°) (Table 2). Thus, by proper combination of the polymers and the number of layers one can obtain a PEM with a required contact angle of any value between 10 and 80°, with either anionic or cationic topmost surface, which may be of potential practical significance.

#### 3.2.3. AFM Characterization of PEMs

- PEM thickness

The PEMs thickness was also measured using the AFM technique. The values obtained are given in Table 3.

The obtained values of PEM thickness found using AFM were very variable and ranged from about 20 to 90 nm. Generally, the swollen films were thicker than the dry ones, and those swollen in 1 M NaCl were thicker than these swollen in PBS, except for C2H&AAm(+) and CAm&AAm(-). Notably, swelling in 1 M NaCl did not result in the disintegration of any PEM indicating their high stability. The values of thickness for swollen 15-layer PEMs (i.e., those with positively charged surface) are qualitatively consistent with those from ellipsometric results, even though for PEMs thicker than 10 nm significant discrepancies may occur between results yielded by both methods [49]. However, for dry PEMs the values of AFM thickness are much lower than those obtained for dry samples using ellipsometric method. This may be attributed to the fact that the ellipsometric measurements of the dry PEMs were conducted immediately after deposition of each layer dried in a stream of nitrogen, so the solvent was much less completely removed than for AFM measurements which were conducted on PEM coatings stored for 48 h at room temperature after being dried with nitrogen. Thus, the solvent content in the dry samples used in AFM was probably much lower than in the samples used in ellipsometric measurements.

- PEM roughness

The RMS roughness of the surface of all tested PEMs was measured, for dry samples and those incubated for 30 min in PBS or in 1 M NaCl (Table 4). The surface of blank sample, i.e., silicon plate after 30 min incubation in piranha solution, were also investigated (Figure S7). The roughness of the substrate was equal to 0.003 ± 0.001 nm, thus the impact of the roughness of the substrate surface on the roughness of tested PEM could be excluded.

The roughness values span a wide range of values – from very smooth dry anionic surface of C2H&AH (RMS of 0.9 ± 0.1 nm) to very rough anionic C1H&Aam (7.0 ± 0.8 nm) or cationic surface of CAm&Aam (7.0 ± 1.1 nm). The general tendencies are that roughness increases in the sequence dry < PBS swollen < 1 M NaCl films (the only three exceptions are positively charged CAm&AH, positively charged C1H&AH, and negatively charged C2H&AAm films). In turn, the PEMs formed with at least one amphiphilic polymer (CAm and/or AAm) are generally rougher than those formed by two hydrophilic polymers (C1H/C2H and AH), with some exceptions, e.g., quite rough cationic surface of C1H&AH in PBS (4.0 ± 0.3 nm) and smooth anionic surface of C2H&AAm in PBS (1.7 ± 0.4 nm).

- Morphology of PEMs

**Figure 4:**
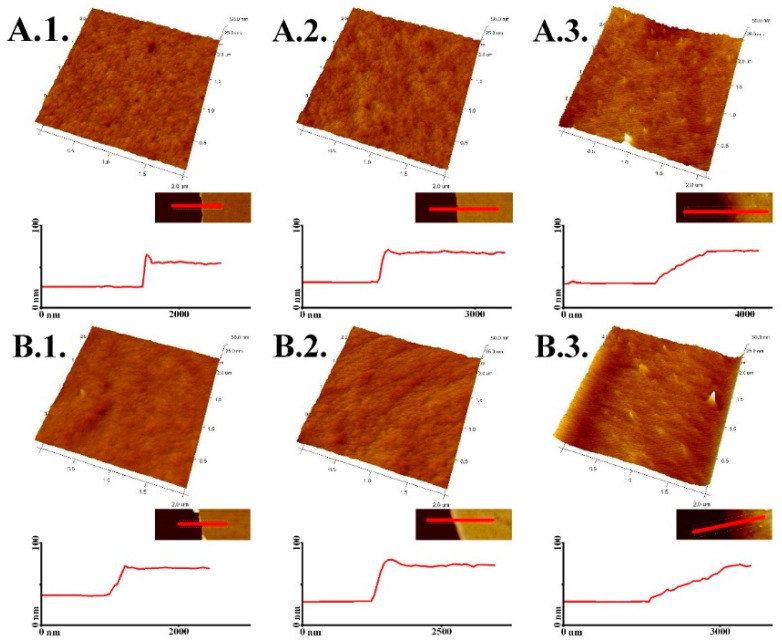
The topography and cross sections of C2H&AH PEMs on silicon for negatively (a panel) and positively charged topmost layer (**B** panel) for dry (**A.1**,**B.1**) and swollen films after 30 min incubation in PBS (**A.2**,**B.2**) or 1 M NaCl solution (**A.3**,**B.3**).

**Figure 5:**
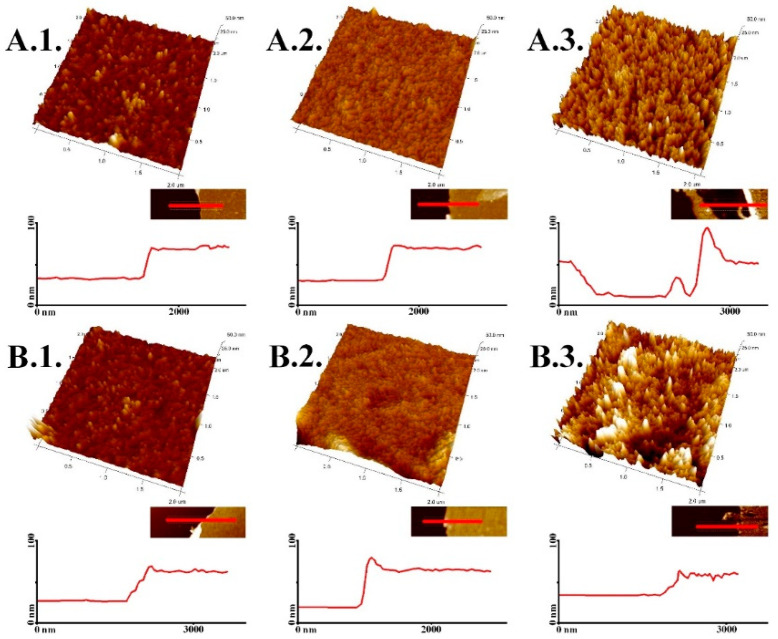
The topography and cross sections of C2H&AAm PEMs on silicon for negatively (**A.1**–**A.3** panel) and positively charged topmost layer (**B.1**–**B.3** panel) for dry (column 1) and swollen films after 30 min incubation in PBS (column 2) or 1 M NaCl solution (column 3).

**Figure 6:**
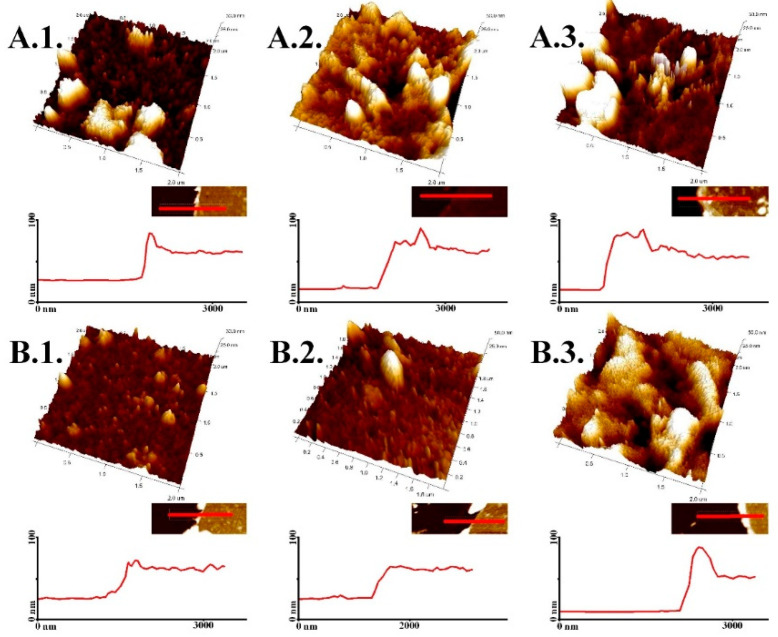
The topography and cross sections of CAm&AAm PEMs on silicon for negatively (**A.1**–**A.3** panel) and positively charged topmost layer (**B.1**–**B.3** panel) for dry (column 1) and swollen films after 30 min incubation in PBS (column 2) or 1 M NaCl solution (column 3).

The morphologies of all obtained PEMs were imaged using AFM (Figure 4, Figure 5 and Figure 6 and Figures S8–S10). The morphologies of C1H&AH PEMs (Figure S8) differed significantly depending on the number of layers and measurement conditions, i.e., they were quite rough with pores homogeneously distributed on the surface for negatively charged dry, negatively- and positively charged PBS-swollen PEMs (RMS roughness of 4.0 ± 0.2, 4.0 ± 0.3, and 4.7 ± 0.4 nm, respectively), while in other cases they are very smooth.

The PEMs of C2H&AH were quite unique compared to PEMs formed by other polymer pairs because they were smooth irrespective of the surface charge and measurement conditions. (Figure 4).

Notably, dry positively charged C2H&AH PEM was the smoothest among all studied systems with RMS roughness of only 0.9 ± 0.1. For this pair of polymers the roughest was the cationic surface of the PEM swollen in 1 M NaCl (RMS roughness of 2.8 ± 0.3), which was also the thickest. This material with thick and loose structure, resistant to dissolution may be useful for preparation of durable polymeric coatings of core-shell objects that may be used for controlled release of active compounds.

The morphology of PEMs formed by polymer pairs in which one or both polymers are amphiphilic was quite different from the previously discussed systems (Figures S9 and S10 and Figure 5 and Figure 6). On the surfaces of these PEMs, objects are observed which may be identified as hydrophobic aggregates formed by hexyl groups. The size of these domains is similar for all the PEMs composed of one amphiphilic polymer (CAm&AH, C1H&AAm, and C2H&AAm, Figures S9 and S10 and Figure 5), while for CAm&AAm these domains are significantly larger (Figure 6), presumably being formed by hydrophobic groups originating from both polymers.

Based on the comparison of the properties (thickness, growth type, wettability, and roughness) of all studied PEMs (Table 5) it may be concluded that the PAH-based polymers may be used for the preparation of PEMs characterized with the required combination of the properties of practical interest.

Dry film of C2H&AAm is rather plain while in solution with higher ionic strength many small spherical objects appear on its surface, presumably aggregates which swell and increase in volume (Figure 5). The material with such properties may be used as a carrier of hydrophobic drugs for controlled delivery systems. Such microdomains may be of a potential interest since they are expected to enhance considerably the loading capacity of the PEMs for incorporation of hydrophobic active compounds, drugs or biomolecules. The polymer aggregates visible on the surface of CAm&AAm are much bigger than those on the surface of C2H&AAm. The formation of aggregates of oppositely charged CAm and AAm polyelectrolytes in solutions of different ionic strengths is currently under investigation in view of their potential application as drug-delivery systems.

## 4. Conclusions

A series of strong anionic and cationic polyelectrolytes, both hydrophilic and amphiphilic, were synthesized based on PAH, the common weak polyelectrolyte. The polymers were carefully characterized and used for preparation of various PEMs. Thickness of the PEMs varied significantly (20–90 nm for 15-layer PEMs). Three types of film growth kinetics were found, i.e., linear, exponential, or mixed exponential-linear, for different PEMs. Contact angle measurements indicated that PEMs of different wettability could be fabricated with contact angle ranging from 10 to 80°. AFM measurements revealed that the PEM surface morphology was variable and could be smooth, porous, or featured with hydrophobic aggregates. The studied PAH derivatives may thus be applied to obtain PEMs with the desired combination of physicochemical properties for practical applications. Due to the fact that the PEMs are stable (insensitive to increased ionic strength) they may be applied in the fabrication of coatings resistant to high ionic strength and/or insensitive to pH changes, e.g., virus concentration and recovery systems for which the virus desorption step requires using high ionic strengths [50], nanofiltration membranes that do not require crosslinking or stabilization [51], desalination membranes [52,53]. Because of the presence of quaternary ammonium functional groups and sulfonate groups, these PEMs may be used for manufacture antimicrobial surfaces as some of the polymers tested here show antibacterial [34] and antiviral properties [54,55]. Other applications, typical of LbL systems, are also possible such as controlled drug delivery [56,57], gene delivery [58] and coatings with controlled cell adhesion properties [59].

## Figures and Tables

**Table 1:** Zeta potential of PAH derivatives in 0.015 M NaCl at 22 °C.

Polymer	Type		ζ* [mV]
	Charge	Hydrophilicity	
C1H	cationic	hydrophilic	+34.7 ± 2.56
C2H	cationic	hydrophilic	+33.2 ± 0.68
CAm	cationic	amphiphilic	+43.7 ± 1.78
AH	anionic	hydrophilic	−42.2 ± 3.80
AAm	anionic	amphiphilic	−43.6 ± 1.48

* The values are averages of 5 measurements with standard deviation.

**Table 2:** The approximate values of water contact angles [°] for all PEMs studied. The values of contact angles for cationic (left) and anionic (right) topmost layers are given for PEMs composed of at least 5 and 6 layers, respectively.

Polycation↓/Polyanion→	AH	AAm
C1H	40/10	50/50
C2H	20/10	60/60
CAm	70/40	80/70

**Table 3:** Thickness of PEMs (14 for negatively and 15 layers for positively charged topmost layer) supported on silicon measured for dry, swollen for 30 min in phosphate-buffered saline (PBS) or 1 M NaCl solution samples.

PEM	PEM Charge *	Dry [nm]	PBS [nm]	1 M NaCl [nm]
C1H&AH	−	21.7 ± 0.3	26.5 ± 0.3	28.0 ± 0.2
	+	26.3 ± 0.4	33.7 ± 0.7	37.3 ± 0.3
C2H&AH	−	29.4 ± 1.0	37.2 ± 1.8	40.9 ± 2.4
	+	34.4 ± 1.4	44.3 ± 1.2	85.1 ± 3.0
CAm&AH	−	46.9 ± 1.5	53.4 ± 2.3	89.9 ± 3.6
	+	52.7 ± 1.3	71.3 ± 3.4	72.8 ± 2.5
C1H&AAm	−	26.8 ± 0.3	32.6 ± 0.5	35.8 ± 0.5
	+	35.1 ± 0.5	37.4 ± 0.7	38.8 ± 0.8
C2H&AAm	−	35.4 ± 2.0	39.6 ± 2.7	39.6 ± 2.3
	+	41.6 ± 1.1	47.7 ± 2.3	25.7 ± 1.2
CAm&AAm	−	33.5 ± 0.7	51.2 ± 1.4	40.9 ± 1.3
	+	40.9 ± 0.6	39.0 ± 1.0	44.1 ± 0.9

* charge of the topmost layer.

**Table 4:** RMS roughness of PEMs supported on silicon. PEMs with negatively and positively charged topmost layer consisted of 14 and 15 layers, respectively. Dry and swollen for 30 min in PBS or 1 M NaCl solution samples were used.

PEM	PEM Charge *	Dry [nm]	PBS [nm]	1 M NaCl [nm]
C1H&AH	−	4.0 ± 0.2	4.0 ± 0.3	2.0 ± 0.1
	+	1.2 ± 0.2	4.7 ± 0.4	1.9 ± 0.0
C2H&AH	−	1.2 ± 0.2	1.4 ± 0.3	2.3 ± 0.3
	+	0.9 ± 0.1	1.1 ± 0.2	2.8 ± 0.3
CAm&AH	−	2.1 ± 0.4	2.7 ± 0.6	3.7 ± 0.7
	+	3.8 ± 0.5	2.9 ± 0.4	2.0 ± 0.1
C1H&AAm	−	4.0 ± 0.7	5.0 ± 0.4	7.0 ± 0.8
	+	5.0 ± 0.2	3.0 ± 0.2	6.6 ± 0.4
C2H&AAm	−	2.6 ± 0.4	1.7 ± 0.4	4.9 ± 0.6
	+	2.6 ± 0.5	2.9 ± 0.5	5.9 ± 0.8
CAm&AAm	−	3.9 ± 0.8	5.9 ± 1.2	3.1 ± 1.0
	+	3.3 ± 0.5	4.3 ± 0.6	7.0 ± 1.1

* charge of the topmost layer.

**Table 5:** Summary of the properties of the PEMs.

PA→ PC↓	Property	AH	AAm
C1H	thickness	thin	thin
	growth type	exponential	exponential/linear
	wettability	fairly hydrophilic/hydrophilic	fairly hydrophilic
	roughness	smooth to medium rough	moderately rough to very rough
C2H	thickness	thick	medium
	growth type	exponential	exponential/linear
	wettability	very hydrophilic	fairly hydrophilic
	roughness	smooth	smooth to rough
CAm	thickness	thick	medium
	growth type	exponential	linear
	wettability	hydrophobic/fairly hydrophilic	hydrophobic to very hydrophobic
	roughness	smooth	rough

## Data Availability

The data presented in this study are available on request from the corresponding authors.

## Supplementary Materials

The following are available online at https://www.mdpi.com/article/10.3390/ma14092361/s1, Figure S1: ATR-FTIR spectra of PAH, C1H, AH, C2H, PAH-Hex, CAm and AAm, Figure S2: ^1^H NMR spectrum of C1H recorded in D_2_O, Figure S3: ^1^H NMR (nuclear magnetic resonance) spectrum of C2H recorded in D_2_O, Figure S4: ^1^H NMR spectrum of CAm recorded in D_2_O, Figure S5: ^1^H NMR spectrum of AH recorded in D_2_O, Figure S6: ^1^H NMR spectrum of AAm recorded in D2O/DMSO-d6 (1:3 *v/v*), Figure S7: The topography and cross sections of silicon surface, after 30 min incubation in piranha solution, used for PEM construction, Figure S8: The topography and cross sections of C1H&AH PEMs on silicon for negatively (A panel) and positively charged topmost layer (B panel) for dry polymeric films (A.1, B.1) and swollen films after 30 min incubation in PBS (A.2, B.2) or 1 M NaCl solution (A.3, B.3), Figure S9: The topography and cross sections of CAm&AH PEMs on silicon for negatively (A panel) and positively charged topmost layer (B panel) for dry and swollen films after 30 min incubation in PBS or 1 M NaCl solution (columns from left to right, respectively), Figure S10: The topography and cross sections of C1H&AAm PEMs on silicon for negatively (A panel) and positively charged topmost layer (B panel) for dry and swollen films after 30 min incubation in PBS or 1 M NaCl solution (columns from left to right, respectively).
