# Supplementary material for: Tuning the Surface Properties of Poly(Allylamine Hydrochloride)-Based Multilayer Films
Source: Materials (Basel). 2021 May 1;14(9):2361. doi: 10.3390/ma14092361 (PMC8125107; doi:10.3390/ma14092361)
Supplement: Supplementary file 1 [file materials-14-02361-s001.zip › materials-1184651-supplementary.pdf]

materials

MDPI

Supplementary Material

Tuning the Surface Properties of Poly(Allylamine Hydrochloride)-Based Multilayer Films

Justyna Ciejka, Michal Grzybala, Arkadiusz Gut, Michal Szuwarzynski, Krzysztof Pyrc, Maria Nowakowska and Krzysztof Szczubiałka

1. Analytical data on the polymers

PAH

^{1}H NMR (400 MHz, D_2O) δ (ppm) = 3.4-2.8 (m, 2H, -*CH$_2$N*-); 2.2-1.9 (m, 1H, -*CH$_2$-CH*); 1.9-1.3 (m, 2H, -*CH$_2$-CH*-).

ATR FT-IR: ν [cm^{-1}]: 3392 ν(N-H, -NH2); 3020 ν(C-H, -CH2-); 2915 ν(N+-H, -NH2 and C-H, -CH2-); 1602 δ(N-H, -NH2); 1508 δ(N-H, -NH2); 1458 δ(C-H, -CH2-, bending); 1385 δ(N-H, -NH2), 1308 δ(C-H, -CH2-, wagging); 1182 δ(C-H, -CH2-, twisting); 1109 δ(N-H, -NH2); 1061 δ(N-H, -NH2, wagging); 1000 ν(C-N); 862 δ(CH2, rocking); 785 δ(N-H, NH2, rocking)

Elemental analysis [%]: C, 33.26; H, 8.93; N, 12.31; C/N, 2.70.

C1H

^{1}H NMR (400 MHz, D_2O) δ (ppm) = 4.0-2.6 (m, 2H, -*CH$_2$N*-); 3.5-3.2 (br s, 1.7H, -*N(CH$_3$)$_3$*); 3.1-2.8 (br s, 1.9H, -*N(CH$_3$)$_2$*); 2.9-2.7 (br s, 2.2H, -*NHCH$_3$*); 2.4-1.9 (m, 1H, -*CH$_2$-CH*-); 1.9-1.1 (m, 2H, -*CH$_2$-CH*-).

ATR FT-IR: ν [cm^{-1}]: 3387 ν(N-H, -NH-); 3020, 2929, 2860 ν(C-H, -CH_2-, -CH_3); 2719; 1606 δ(N-H, -NH-); 1468 δ(C-H, -$N^+(CH_3)_3$); 1177 δ(C-H, -CH_2-, twisting); 1132; 961; 913.

Elemental analysis [%]: C, 42.47; H, 9.57; N, 11.02; C/N, 3.86.

C2H

^{1}H NMR (400 MHz, D_2O) δ (ppm) = 4.6-4.2 (m, 1H, -*CH$_2$-CH(OH)-CH$_2$*-); 3.8-3.4 (m, 2H, -*CH$_2$N(CH$_3$)$_3$*); 3.4-3.2 (m, 9H, -*N(CH$_3$)$_3$*); 3.1-2.2 (m, 4H, -*CH$_2$NHCH$_2$*-); 2.1-0.8 (m, 3H, -*CH$_2$-CH*-).

ATR FT-IR: ν [cm^{-1}]: 3352 ν(N-H, -NH-); 3259 ν(O-H); 3020, 2919, 2850 ν(C-H, -CH_2-, -CH_3); 1641 cm^{-1} δ(N-H, -NH-); 1478 δ(C-H, -$N^+(CH_3)_3$); 1144; 1096 δ(O-H, -R(OH)-); 964 δ(O-H); 919; 871

Elemental analysis [%]: C, 46.13; H, 9.94; N, 11.29; C/N, 4.09.

AH

^{1}H NMR 400 MHz, D_2O) δ (ppm) = 3.2-2.6 (m, 2H, -*CH$_2$N*-); 2.0-1.6 (m, 1H, -*CH$_2$-CH*-); 1.6-0.7 (m, 2H, -*CH$_2$-CH*-).

ATR FT-IR: ν [cm^{-1}]: 3448, 3266 ν(N-H, -NH_2, -NH-); 2921, 2858 ν(C-H, -CH_2-, -CH_3); 1640 cm^{-1} δ(N-H, -NH-); 1433 δ(C-H, -CH_2-, bending); 1169 ν_{as}(S=O, -N-SO_3^-); 1032 ν_s(S=O, -N-SO_3^-)

Elemental analysis [%]:C, 21.62; H, 4.60; N, 8.14; S, 25.93; C/N, 2.66; S/N, 3.19.

PAH-Hex

ATR FT-IR: ν [cm^{-1}]: 3380 ν(N-H); 2952, 2927, 2858 ν(C-H, -CH_2-, -CH_3); 1602 δ(N-H, -NH_2); 1512 δ(N-H); 1460 δ(C-H, -CH_2-, bending); 1378 δ(N-H); 1012 ν(C-N); 723.

Elemental analysis [%]: C, 55.27; H, 10.71; N, 7.47; C/N, 7.40.

CAm

^{1}H NMR: (400 MHz, D_2O) δ (ppm) = 4.2-4.5 (m, 1H, -*CH$_2$-CH(OH)-CH$_2$*-); 3.3-3.1 (m, 11H, -*N(CH$_3$)$_3$* and -*CH$_2$-N(CH$_3$)$_3$*); 3.0-2.2 (m, 13.3H, -*CH$_2$NH*- and -*NHCH$_2$*-); 2.2-1.0 (m, 38.8H, -*CH$_2$-CH*- and -*(CH$_2$)$_4$CH$_3$*); 1.0-0.6 (br s, 8.9H, -*CH$_3$*).

ATR FT-IR: ν [cm^{-1}]: 3368 ν(N-H, -NH-); 3271 ν(O-H), 2955, 2921, 2854 ν(C-H, -CH_2-, -CH_3); 2806; 1641 cm^{-1} δ(N-H, -NH-); 1462 δ(C-H, -CH_2-, bending); 1376; 1305; 1212; 1103 δ(O-H, -R(OH)-, broad band); 1019 ν(C-N); 970; 916; 726.

Elemental analysis [%]: C, 58.50; H, 11.28; N, 9.61; C/N, 6.09.

AAm

^{1}H NMR: (400 MHz, D_2O) δ (ppm) = 3.0-2.6 (m, 26.6H, -*C**H**$_2$NH*- and -*NHC**H**$_2$*-); 1.8-1.0 (m, 14.5H, -*C**H**$_2$-C**H***- and -*(C**H**$_2$)$_3$*-); 0.9-0.7 (m, 5H, -*C**H**$_2$C**H**$_3$*).

ATR FT-IR: ν [cm^{-1}]: 3435 ν(N-H, -NH_2, -NH-); 3268; 2955, 2926, 2857 ν(C-H, -CH_2-, -CH_3); 1641 cm^{-1} δ(N-H, -NH-); 1462 δ(C-H, -CH_2-, bending); 1376 δ(N-H); 1303, 1219 δ(C-H, -CH_2-); 1164 ν_{as}(S=O, -N-SO_3^-); 1031 ν_s(S=O, -N-SO_3^-); 983; 888 δ(CH_2, rocking); 795 δ(NH_2, rocking); 726.

Elemental analysis [%]: C, 40.76; H, 7.52; N, 6.82; S, 21.18; C/N, 5.98; S/N, 1.79.

2. FT-ATR-IR analysis of PAH derivatives

The ATR-FT-IR spectra of PAH and its five strongly charged derivatives, i.e. C1H, C2H, AH, CAm, AAm (and a hydrophobic intermediate PAH-Hex), are shown in **Figure S1**. In the spectrum of PAH, the broad absorption band at approximately 3392 cm^{-1} was attributed to the stretching vibration of the N-H bond in the amine group. The symmetric and asymmetric vibrations of this group were found at 1602 and 1385 cm^{-1}, respectively. The deformation vibrations of the amine group were found at 1508, 1109, 1061, and 785 cm^{-1}. The very broad band from 3150 to 2400 cm^{-1}, with maximum around 2915 cm^{-1}, was assigned to the stretching vibrations of C-H in methylene group, which overlapped with the band of N-H in protonated primary amine groups. The bands at 1458, 1308, 1182, and 862 cm^{-1} correspond to deformation vibrations of C-H in methylene groups. Finally, the C-N absorption band of PAH is located at 1000 cm^{-1}.

The FT-ATR-IR spectrum of hydrophobic intermediate PAH-Hex exhibits two additional adsorption bands which appear at 2952 and 2858 cm^{-1}, and along with the maximum at 2927 cm^{-1} form characteristic "triple" band also found in FT-ATR-IR spectra of amphiphilic polyelectrolytes CAm and AAm. This is attributed to stretching vibrations of C-H in the methylene and methyl groups. Moreover, very broad band spanning from 3150 to 2400 cm^{-1} suggests, that primary and secondary amine groups of PAH-Hex are protonated. In addition, the C-N stretching band is slightly shifted to 1012 cm^{-1}. These changes confirm a covalent attachment of *n*-hexyl chains to the amine PAH groups.

In the FT-ATR-IR spectra of both hydrophilic (AH) and amphiphilic (AAm) *N*-sulfonated PAH derivatives, the presence of two characteristic -N-SO_3^- bands at 1164 and 1031 cm^{-1} confirms sulfonation of amine groups. These bands correspond to antisymmetric and symmetric stretching vibrations of S=O bond, respectively, giving the unambiguous proof of the presence of sulfoamino groups in AH and AAm polymers.

In the FT-ATR-IR spectra of GTMAC-derivatives of PAH (C2H and CAm), three new absorption bands appeared at ~3260, ~1100, and 964 cm^{-1}. The first, very broad band represents the combined stretching vibration of O-H and N-H bonds. Two other bands correspond to the deformation vibrations of the hydroxyl group of GTMAC. The band at 964 cm^{-1} may be attributed to the characteristic band of C-N in quaternary ammonium groups. This band is also present in FT-ATR-IR spectrum of C1H, thus it confirms the presence of quaternary ammonium groups in both C2H and C1H. Finally, in FT-ATR-IR spectra of C2H, a new strong band at 1478 cm^{-1} characteristic of GTMAC-modified polymers was identified as -N^+-$(CH_3)_3$ group stretching derived from GTMAC substituent. This band is barely visible in FT-ATR-IR spectrum of CAm, probably due to the relatively low degree of substitution quaternary ammonium groups, i.e. 28%, compared to C2H, which was fully quaternized. Additionally, it is shifted to 1462 $cm^{-1,}$ probably because of overlapping with deformation vibrations of C-H in hydrocarbon chain. Interestingly, such band was also found in FT-ATR-IR spectrum of C1H, slightly shifted to 1468 cm^{-1}, thus it may be also assigned to quaternary ammonium groups of methylated polymer.

Noteworthy, C2H, AH, CAm, and AAm proved to be hygroscopic. Therefore, in the FT-ATR-IR spectra of these polymers, very broad absorption bands in 3000-3500 cm^{-1} region were observed. Furthermore, the band of O-H deformation vibrations in water molecules was also observed at 1640 cm^{-1}, which overlapped with N-H symmetric vibrations of amine groups of PAH derivatives.

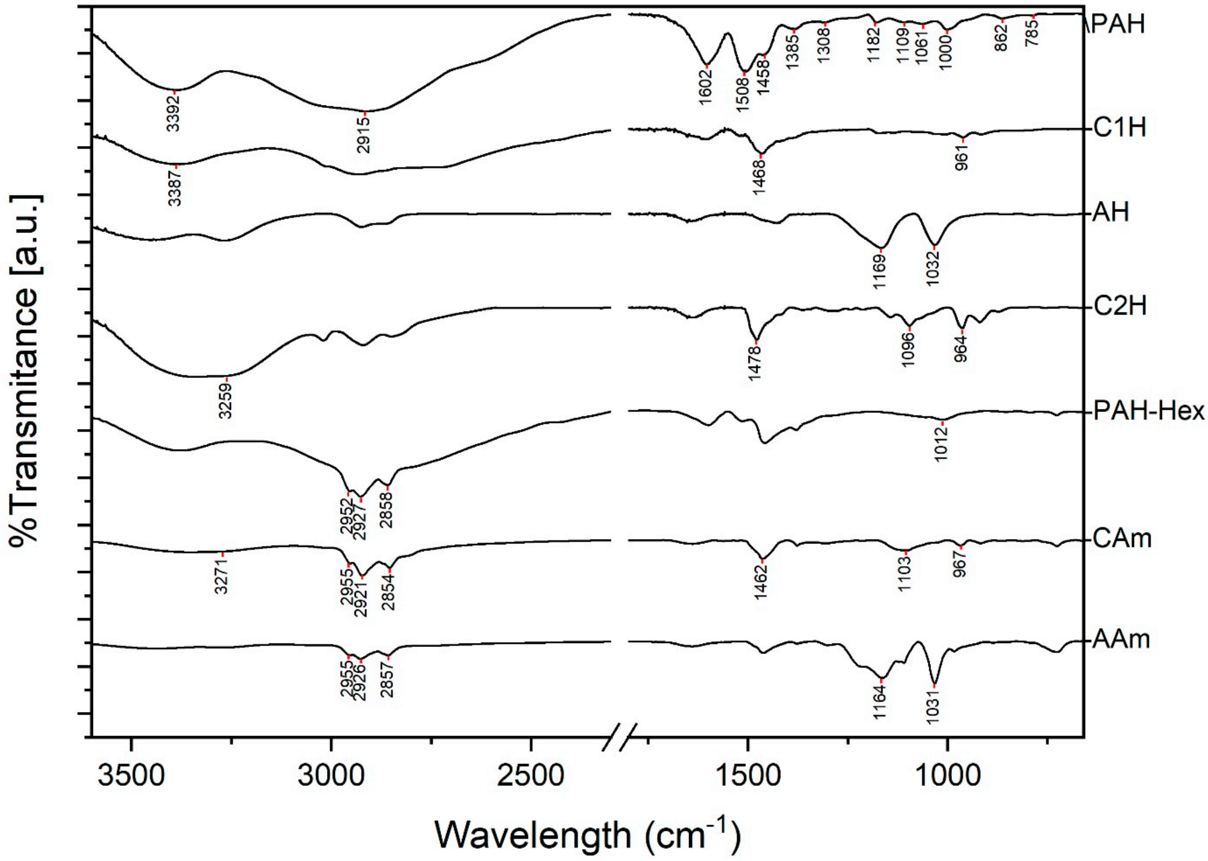

Figure S1. ATR-FTIR spectra of PAH, C1H, AH, C2H, PAH-Hex, CAm and AAm.

3.1. H NMR analysis of PAH derivatives

In order to estimate the degree of substitution of PAH with different groups ^{1}H NMR analysis was performed. In ^{1}H NMR spectrum of C1H a group of broad resonance signals spanning from 1.2 to 2.4 ppm was identified and unambiguously assigned to the main chain -*CH₂*-*CH*- (3H) protons (**Figure S2**). Downfield shifted region of the spectrum includes an overlapping group of signals (from 2.5 to 3.8 ppm), which was attributed to superimposed resonance lines of -CH_3 protons (in mono-, di- and tri-methylated amine groups, respectively) along with broad signal from independent -CH_2N- (2H) moieties. For the purpose of the estimation of methylation degree, spectrum deconvolution was employed. An accurate Gauss function fitting, resulted in a separation of overlapped region, facilitating an integration of diagnostic signals, which were referred to a normalized (3H) integration of -CH_2-*CH*- PAH counterpart. These calculations proved that methylation reaction of PAH occurs on each amine group present in the structure of polymer and the content of mono-, di-, and tri-methylated amine groups was ca. 46%, 30%, and 24%, respectively.

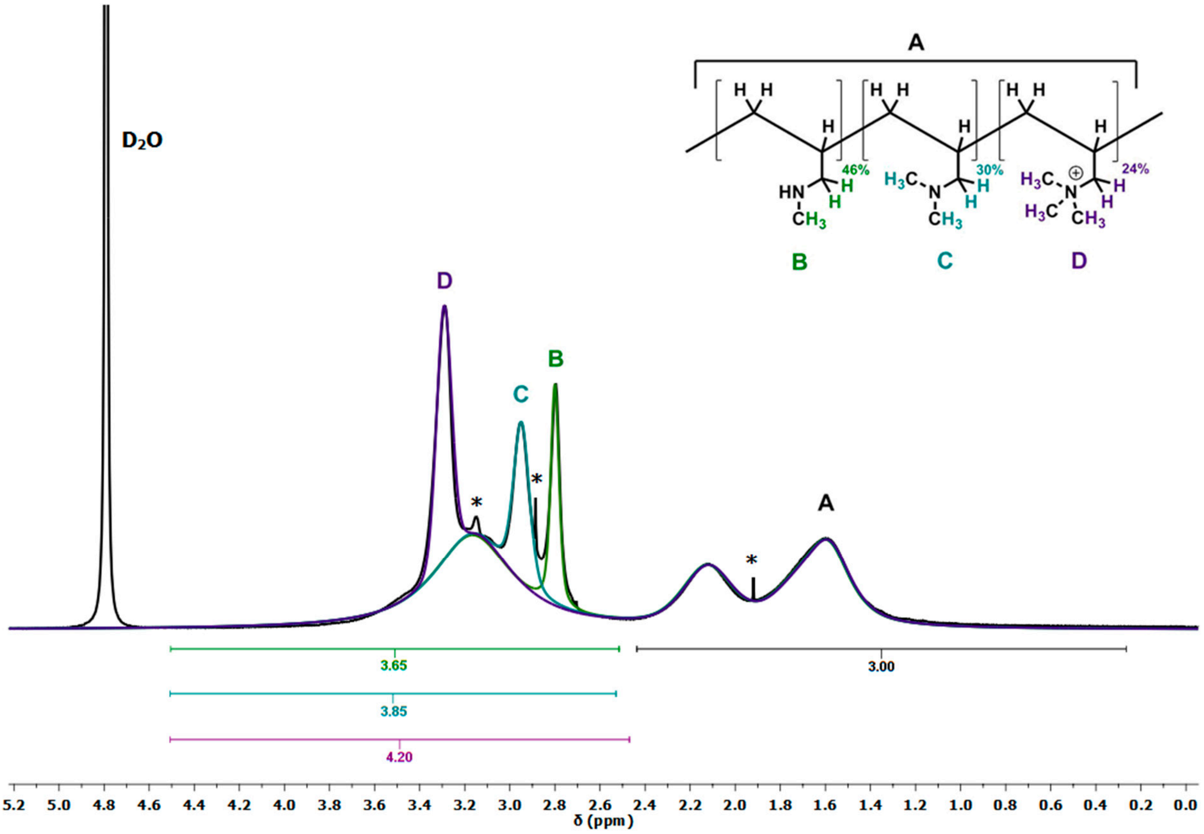

Figure S2. ^{1}H NMR spectrum of C1H recorded in D_2O.

The structure of **C2H** was unambiguously confirmed by ^{1}H NMR experiment. The recorded spectrum consist of five well-separated resonance signals at 0.8 - 2.1 ppm, 2.2 - 3.1 ppm, 3.1 - 3.3 ppm, 3.4 - 3.8 ppm and 4.2 - 4.6 ppm, which could be clearly attributed to -*C**H**$_2$-C**H**-* (3H), -*C**H**$_2$NHC**H**$_2$-* (4H), -*N(C**H3**)3* (9H), -*C**H**$_2$N(CH$_3$)$_3$* (2H) and -*CH$_2$-C**H**(OH)-CH$_2$-* (1H) counterparts, respectively (**Figure S4**). Moreover, the ratio of signal integrations referred to main chain fragment -C**H**$_2$-C**H**- (3H) suggests that all the amine groups were substituted with GTMAC (DS = 100%).

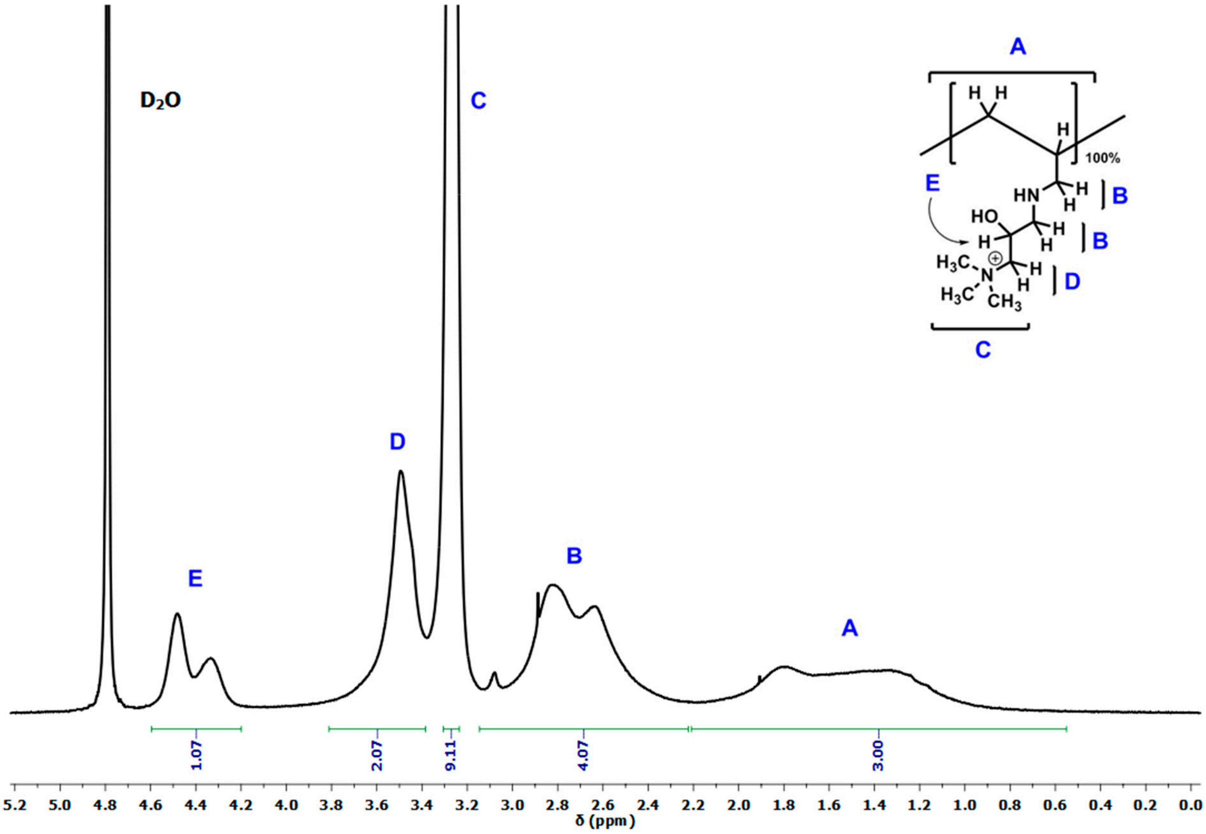

Figure S3. ^{1}H NMR spectrum of C2H recorded in D_2O.

^{1}H NMR spectrum of **CAm** indicated an array of resonance signals, characteristic of both GTMAC and hydrophobic *n*-hexyl motifs present in the structure (**Figure S5**). The most upfield shifted signal located at 0.7 - 0.9 ppm was assigned to terminal methyl protons in the hexyl group. On the other hand, the signal at 4.2 - 4.5 ppm region was undoubtedly attributed to *-CH₂-C**H**(OH)-CH₂-* motif confirming the presence of GTMAC moiety. The resolved integrations of both signals were used as diagnostic values for estimation of the degree of substitution, referring to an area under overlapping resonance lines (at 0.6 - 2.2 ppm) originating from *-(C**H**₂)₄-* (hydrophobic part) and *-C**H**₂-C**H**-* (main chain part) protons. These calculations proved that CAm contains ca. 13%, 59% and 28% of unsubstituted, N-hexylated and GTMAC-modified amine groups, respectively.

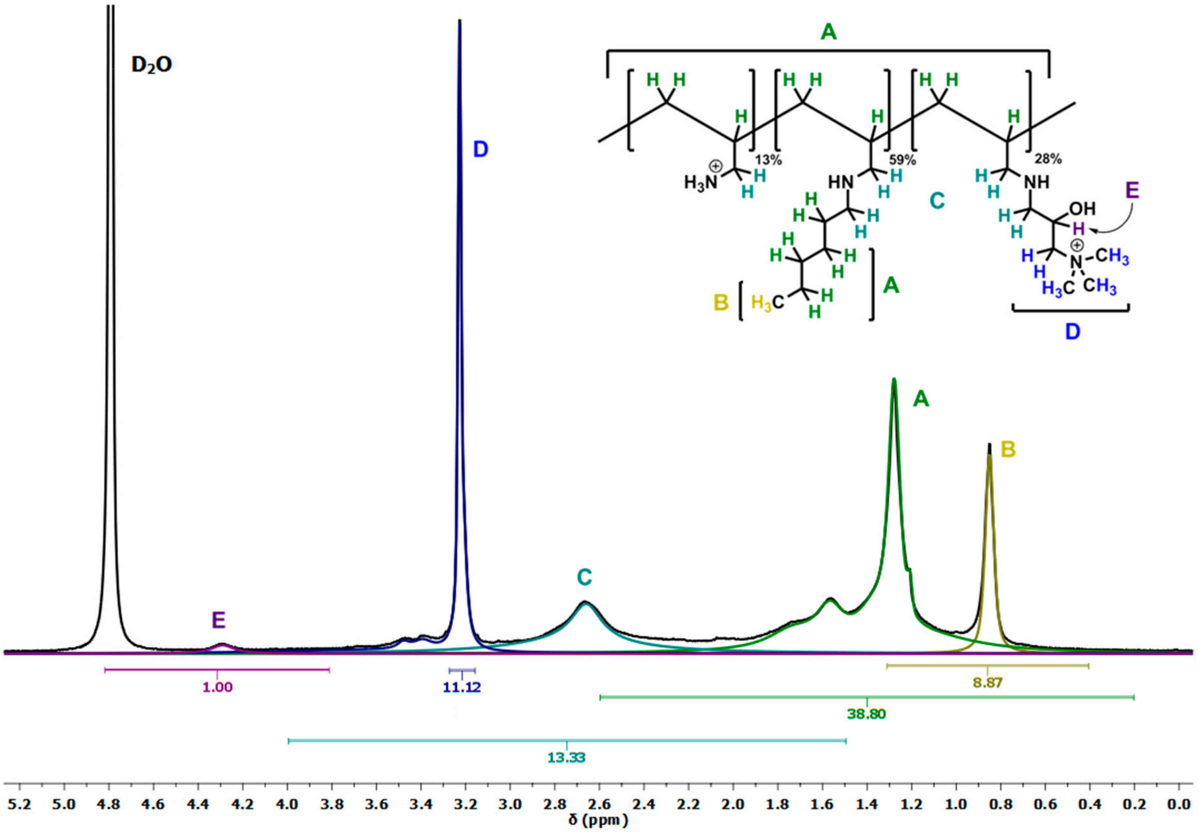

Figure S4. ^{1}H NMR spectrum of CAm recorded in D_2O.

In the upfield region of the ^{1}H NMR spectrum of AH, a group of signals located between 0.8 ppm to 2.0 ppm was assigned as resonance signals originating from main chain -*CH*₂-*CH*- (3H) protons (**Figure S3**). Furthermore, downfield shifted signals at 2.6 – 3.2 ppm were attributed to a superimposed spectral lines derived from magnetically unequal -*CH*₂*N*- (2H) repeat units along with a residual trimethylamine (TMA) signal. Surprisingly, the deconvolution of this diagnostic signal revealed the presence of three Gaussian components. Comparison of each independent integral, referred to a normalized (3H) integration of -*CH*₂-*CH*- PAH counterpart, provided an assessment of the degree of substitution. These calculations suggest that AH contains ca. 44% of unsubstituted (signal at 2.8 ppm), ca. 21% of di-sulfonated (signal at 3.0 ppm), and ca. 35% of mono-sulfonated (signal at 3.1 ppm) amine groups.

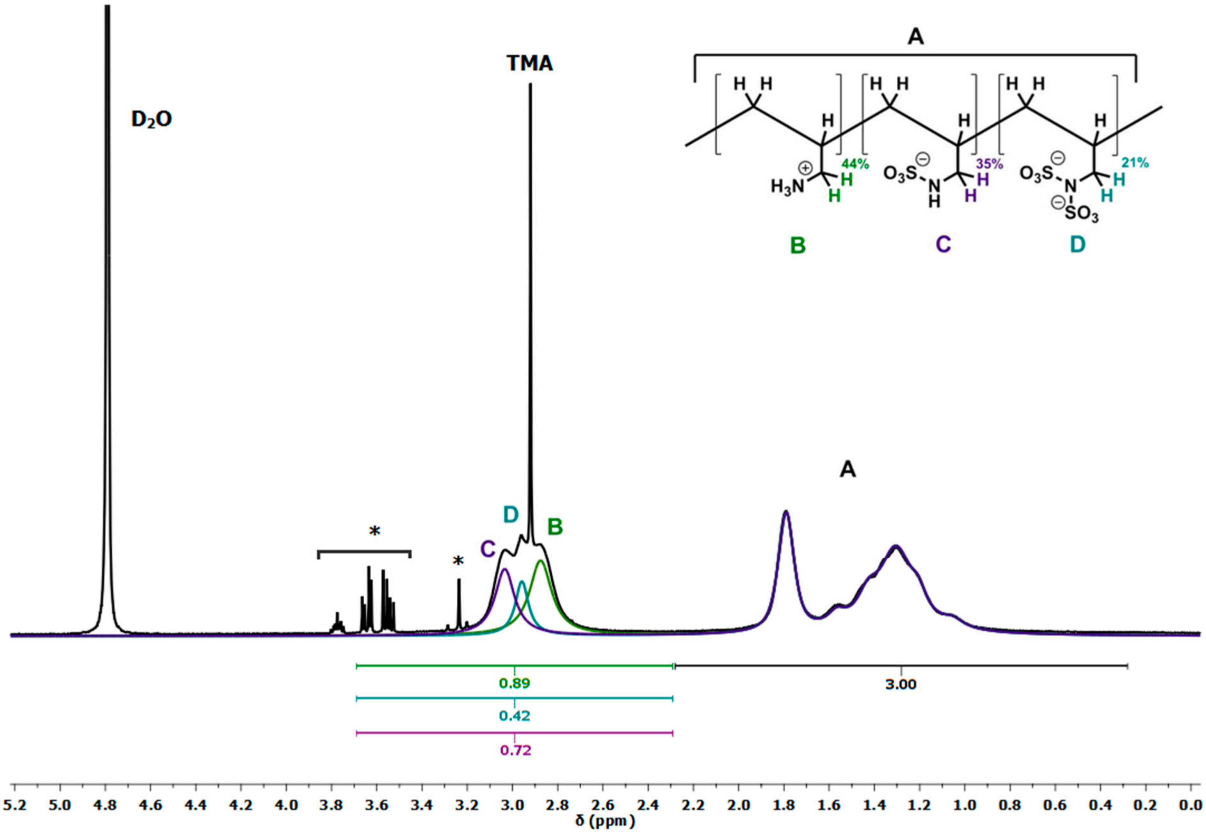

Figure S5. ^{1}H NMR spectrum of AH recorded in D_2O.

In the ^{1}H NMR spectrum of **AAm** the resonance signal spanning from 0.7 to 0.9 ppm was identified and assigned to a hydrophobic terminal - *CH₂CH₃* (5H) residue. To estimate the degree of substitution, a fixed integration of this spectral range was referred to the area under region located at 1.0 - 1.8 ppm, which was attributed to a superimposed signal of main chain -*CH2*-*CH*- (3H) protons with -$(CH_2)_3$- protons of *n*-hexyl motif (**Figure S6**). These calculations suggest that **AAm** contains ca. 35% of N-hexylated amine groups. Furthermore, an intense sharp resonance line at 2.8 ppm was assigned to overlapping signals from -*CH₂N*- and -NC**H₂**- protons. However, high intensity and uneven shape of this signal does not allow an accurate deconvolution of this spectral region and the assessment of N-sulfonation degree. Surprisingly, elemental analysis of **AAm** revealed, that hydrophobization degree of polymer reached up to 60%, as C/N ratio was estimated at 5.98. This result is fully consistent with both ^{1}H NMR and elemental analysis performed for **PAH-Hex**, which was used as a starting material for sulfonation reaction.

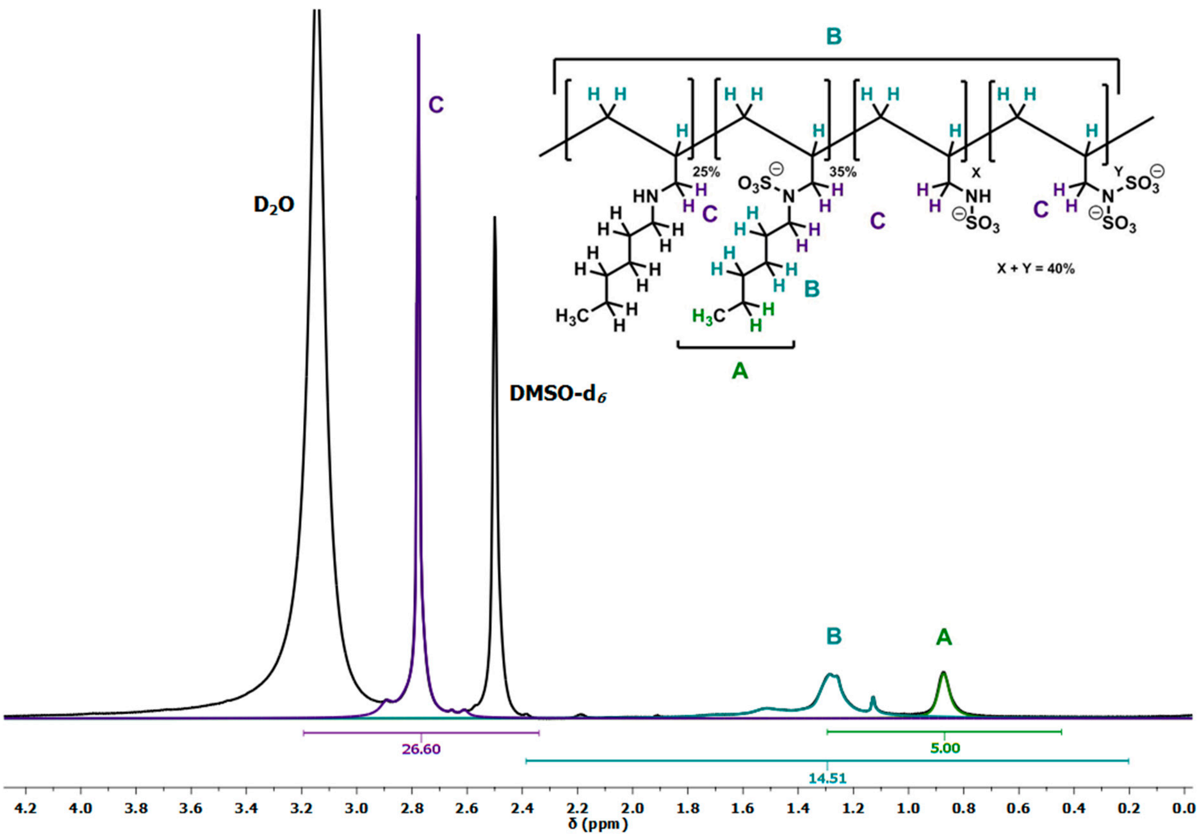

Figure S6. ^{1}H NMR spectrum of AAm recorded in D_2O/DMSO-d_6 (1:3 v/v).   .

4. AFM analysis of PEMs

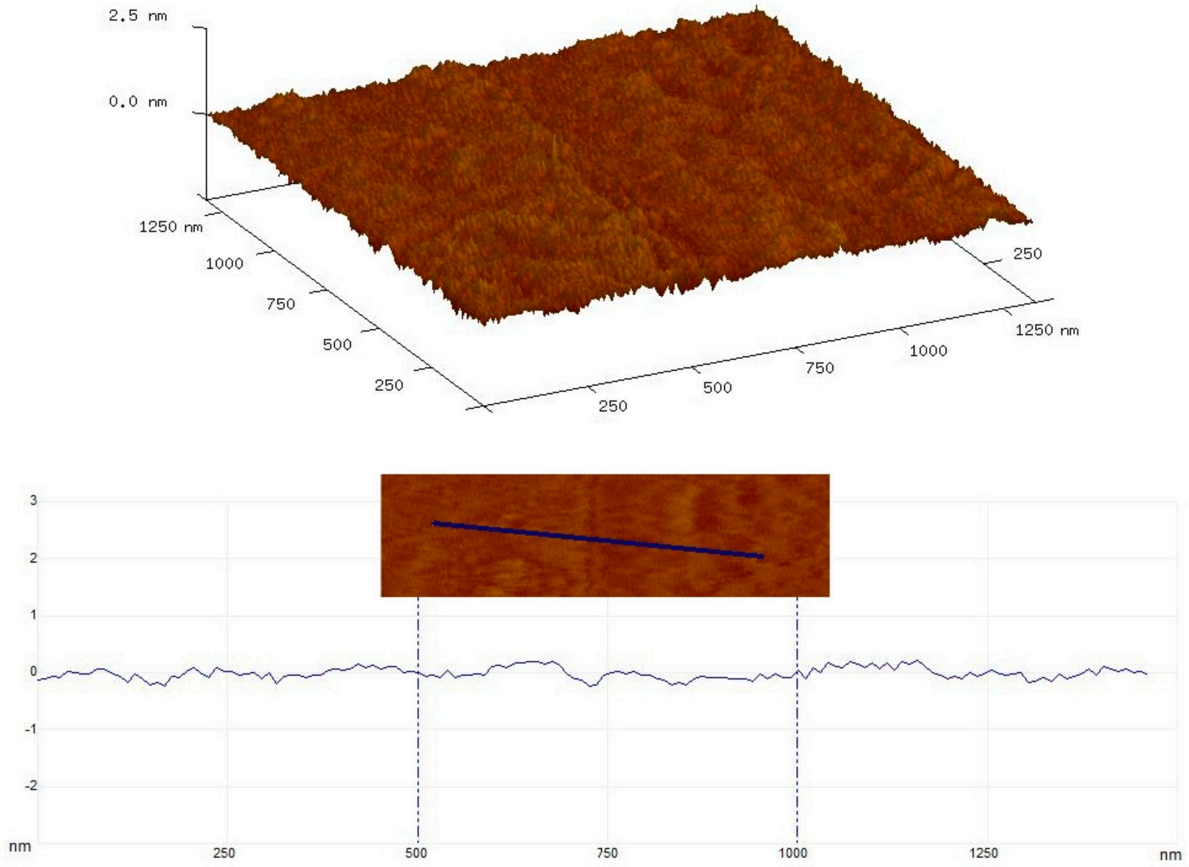

Figure S7. The topography and cross sections of silicon surface, after 30 min incubation in piranha solution, used for PEM construction.

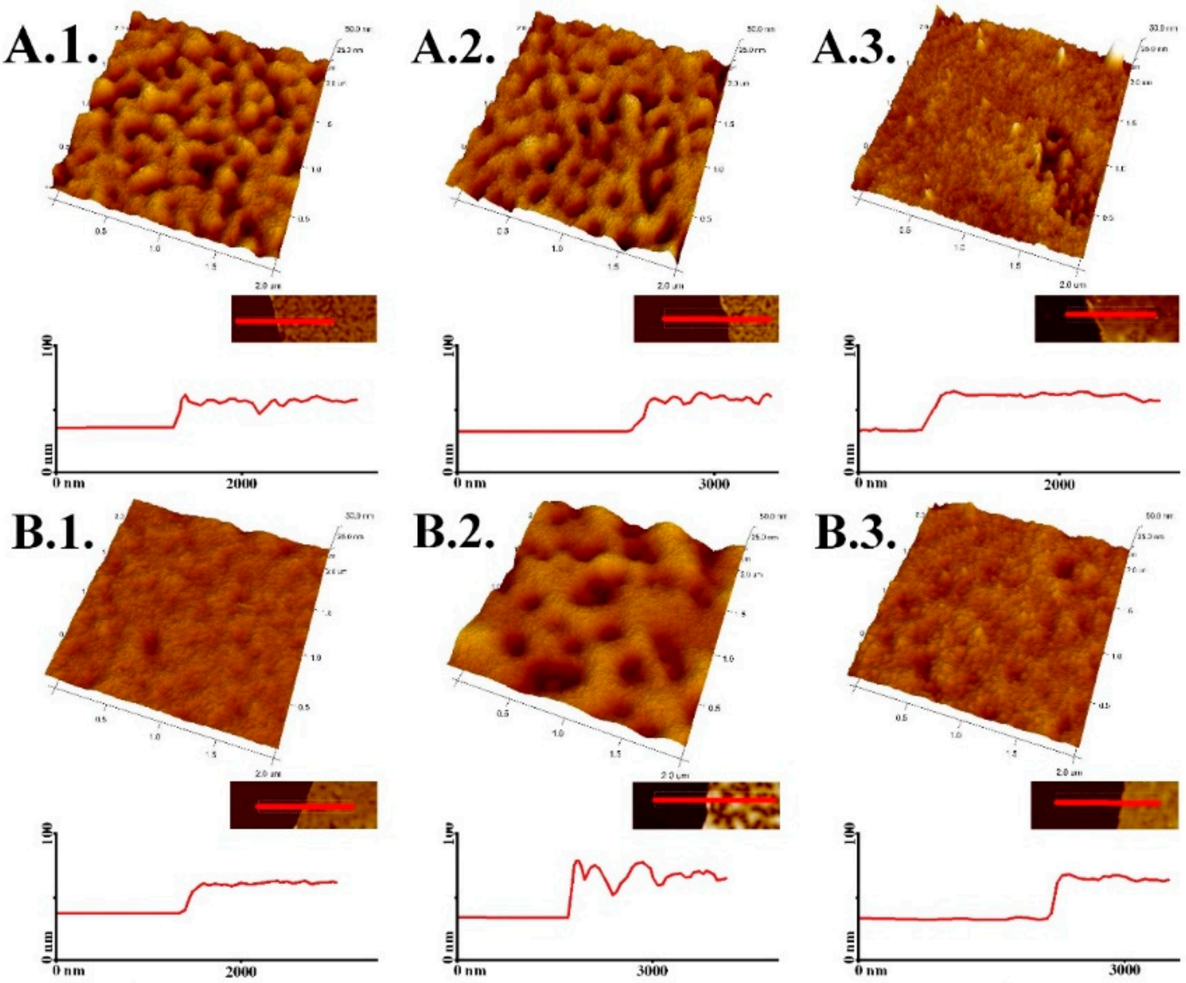

Figure S8. The topography and cross sections of C1H&AH PEMs on silicon for negatively (A panel) and positively charged topmost layer (B panel) for dry polymeric films (A.1, B.1) and swollen films after 30 min incubation in PBS (A.2, B.2) or 1 M NaCl solution (A.3, B.3).

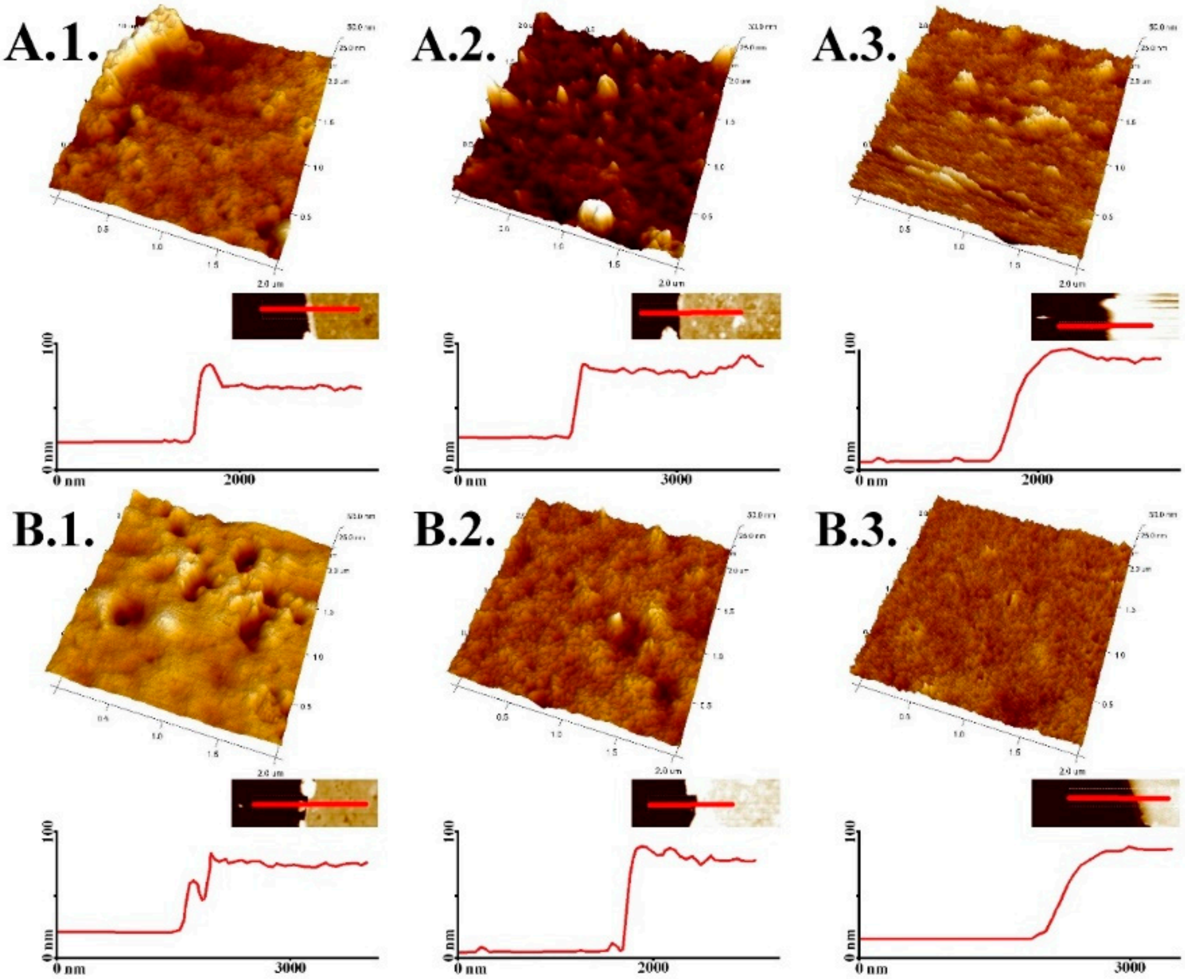

Figure S9. The topography and cross sections of CAm&AH PEMs on silicon for negatively (A panel) and positively charged topmost layer (B panel) for dry and swollen films after 30 min incubation in PBS or 1 M NaCl solution (columns from left to right, respectively).

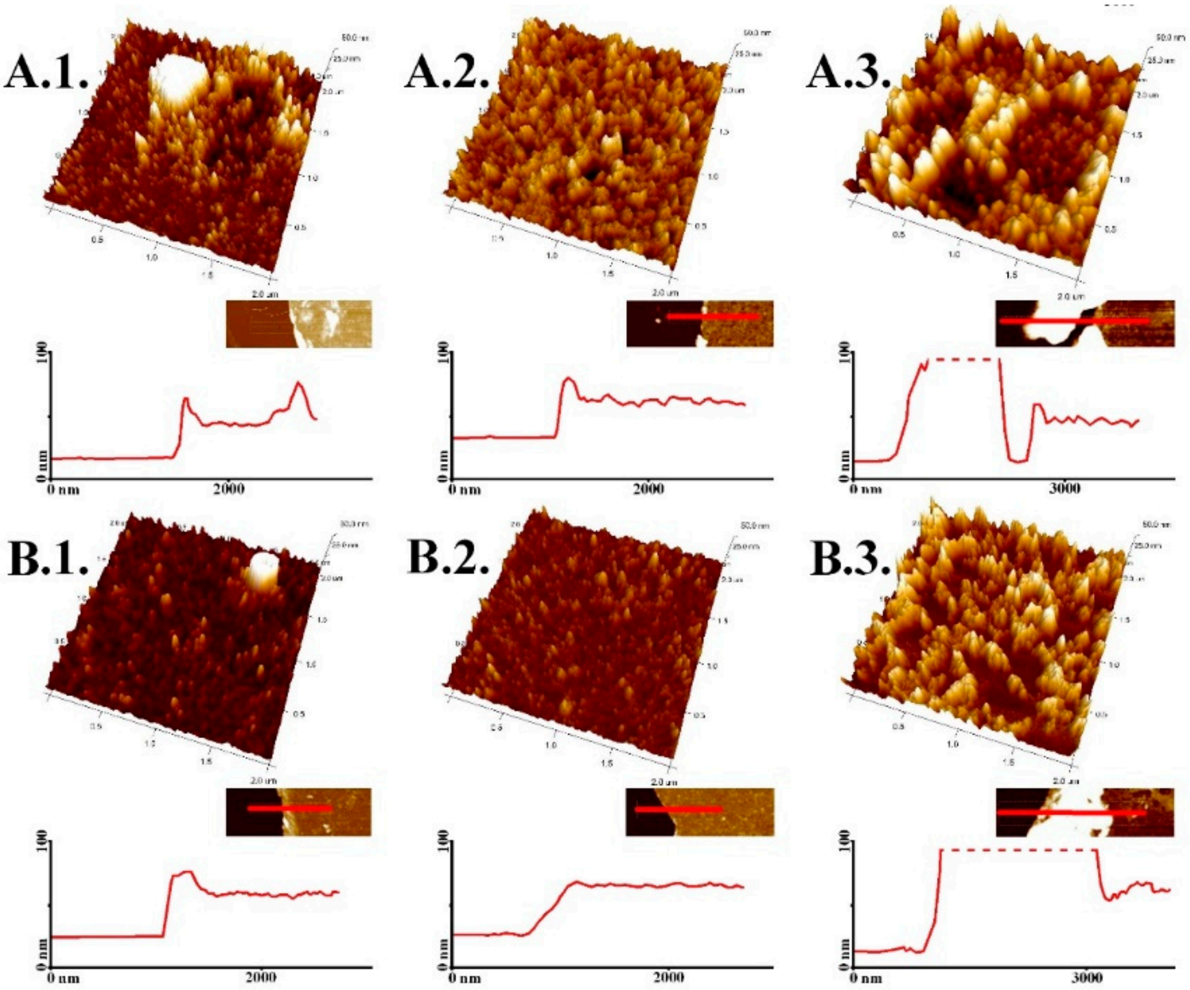

Figure S10. The topography and cross sections of C1H&AAm PEMs on silicon for negatively (A panel) and positively charged topmost layer (B panel) for dry and swollen films after 30 min incubation in PBS or 1 M NaCl solution (columns from left to right, respectively).
